# Proteomic and histopathologic profiling reveal molecular features and clinical biomarkers of coronary atherosclerosis

**DOI:** 10.1186/s40364-025-00846-3

**Published:** 2025-10-22

**Authors:** Xinjie Xu, Zhongli Chen, Sifei Chen, Jiansong Huang, Jiali Chen, Jiaying Cao, Hang Gao, Enhao Huang, Yibo Zhang, Xiangjie Li, Yifeng Zhang, Xiaorui Liu, Shengkang Huang, Ke Yang, Yang Yang, Wenjia Zhang, Ying Song, Liang Chen, Zhan Hu

**Affiliations:** 1 State Key Laboratory of Cardiovascular Disease, Fuwai Hospital, National Center for Cardiovascular Diseases, Chinese Academy of Medical Sciences, Peking Union Medical College, Beijing, China; 2 The First School of Clinical Medicine, Nanfang Hospital, Southern Medical University, Guangzhou, China; 3 The Second School of Clinical Medicine, Zhujiang Hospital, Southern Medical University, Guangzhou, China; 4 Department of Cardiology, Ruijin Hospital, Shanghai Jiao Tong University School of Medicine, Shanghai, China

**Keywords:** Proteomics, Histopathologic classification, Human coronary artery, Atherosclerosis, Biomarker

## Abstract

The molecular features of coronary atherosclerosis progression remain incompletely understood. A comprehensive characterization of coronary proteome dynamics during atherosclerosis progression could facilitate the identification of novel biomarkers for early detection of plaque initiation and risk assessment of plaque destabilization. We performed proteomics on human coronary artery specimens representing five progressive histopathologic stages of atherosclerosis according to the modified AHA classification, including adaptive intimal thickening (AIT), pathological intimal thickening (PIT), fibroatheroma (FA), thin cap fibroatheroma (TCFA), and ruptured plaque (RP). The results revealed progressive dysregulation of complement and coagulation cascades and extracellular matrix (ECM) organization during histopathologic progression, particularly in plaque initiation and destabilization. Integrated single-cell RNA sequencing data showed that complement and coagulation pathways were predominantly upregulated in fibroblasts and macrophages, while ECM organization was elevated in fibroblasts and smooth muscle cells. Plasma proteomics in a discovery cohort identified THBS1, ECM2, and C1R proteins as robust diagnostic biomarkers from among the overlapping complement and ECM proteins found in the tissue proteomics. The combination of these biomarkers achieved area under the curve (AUC) values of 0.831 in the training set and 0.764 in the test set for identifying coronary artery disease (CAD). In both the discovery cohort and the external validation cohort, this biomarker panel distinguished stable CAD from non-stenosis controls (AUC: 0.765 and 0.841, respectively) and identified ACS patients (AUC: 0.786 and 0.822, respectively). These findings elucidate the proteomic landscape of atherosclerosis progression and establish a three-protein biomarker panel with potential for CAD diagnosis.

**Supplementary Information:**

The online version contains supplementary material available at 10.1186/s40364-025-00846-3.

**To the editor**,

Coronary atherosclerosis is a major driver of global cardiovascular mortality [1]. However, proteomic changes across plaque progression stages remain poorly characterized, limiting early detection and risk assessment of plaque destabilization. Therefore, we performed histopathologic profiling and proteomics on 20 human coronary segments across different pathological stages [adaptive intimal thickening (AIT), pathological intimal thickening (PIT), fibroatheroma (FA), thin cap fibroatheroma (TCFA), and ruptured plaque (RP)] (Fig. 1A, Fig. S1A, Table S1). Weighted gene co-expression network analysis identified 12 modules that were significantly altered during atherosclerosis progression. Among these, complement activation (M5: *r* = 0.72, *P* = 3.7 × 10⁻⁴) and extracellular matrix (ECM) remodeling (M3: *r* = 0.61, *P* = 4.4 × 10⁻³) showed the strongest correlations with atherosclerosis, and their module scores were significantly increased during histopathologic progression (Fig. 1B and C, Fig. S1B-S1C, Table S2). Partial least squares discriminant analysis (PLS-DA) demonstrated progressive alterations from AIT, PIT, FA to TCFA and RP stages (Fig. 1D). The number of significant differential expression proteins (DEPs) at each stage compared to AIT was increased with the progression of atherosclerosis (Fig. 1E, Fig. S1E-S1H and Table S3). Complement and ECM organization pathways ranked the top in enrichment analysis of each stage versus AIT DEPs and stage-shared DEPs (Fig. 1F, Fig. S1D and Fig. S1I-S1L). Previous proteomics data on human coronary arteries and carotid plaques also validated severe dysregulation of complement and ECM pathways in atherosclerosis (Figure S2A-S2D) [2–5]. Single sample enrichment score showed complement and ECM organization activities were increased continuously and positively correlated with each other (*r* = 0.92, *P* = 4.3 × 10⁻⁴) (Fig. 1G and H). K-means clustering of DEPs identified four clusters. Cluster 1 showed a progressive increase throughout atherosclerosis progression and was enriched in complement and coagulation cascades. Cluster 2 spiked at the TCFA stage and was enriched in complement and neutrophil degranulation. Cluster 3 was elevated at the PIT stage and remained stable; it was enriched in ECM organization. Cluster 4 decreased continuously and was enriched in actin and cytoskeleton-related pathways (Fig. 1I-L). Specifically, classical complement pathway activation (C1QB/C1QC) was upregulated in PIT, while alternative pathway components (C3, C5-9) peaked in TCFA/RP (Fig. S3 and Table S4). Collagens (COL1A1, COL4A1, COL5A1) were elevated in PIT/FA but declined in TCFA, accompanied by upregulation of matrix metalloproteinases (MMP7/MMP12) (Table S5). These results suggested sustained dysregulation of complement activation and ECM organization during atherosclerosis histopathologic progression. We integrated human coronary single-cell RNA sequencing (scRNA-seq) data and mapped these pathway proteins to cellular subtypes (Fig. S4A-S4B) [6–11]. Complement signaling was predominantly localized at fibroblasts and macrophages, while ECM organization centered on FB and vascular smooth muscle cells (VSMCs) (Fig. S4C-S4D). Along the pseudo-time trajectory of monocyte-to-macrophage differentiation, we observed a progressive elevation in complement pathway activity (Fig. 1M and N, Fig. S4E-S4F). VSMC-to-FB transition drove a parallel increase in the expression of complement and ECM organization proteins (Fig. 1O and P, Fig. S4G-S4I).

**Fig. 1:**
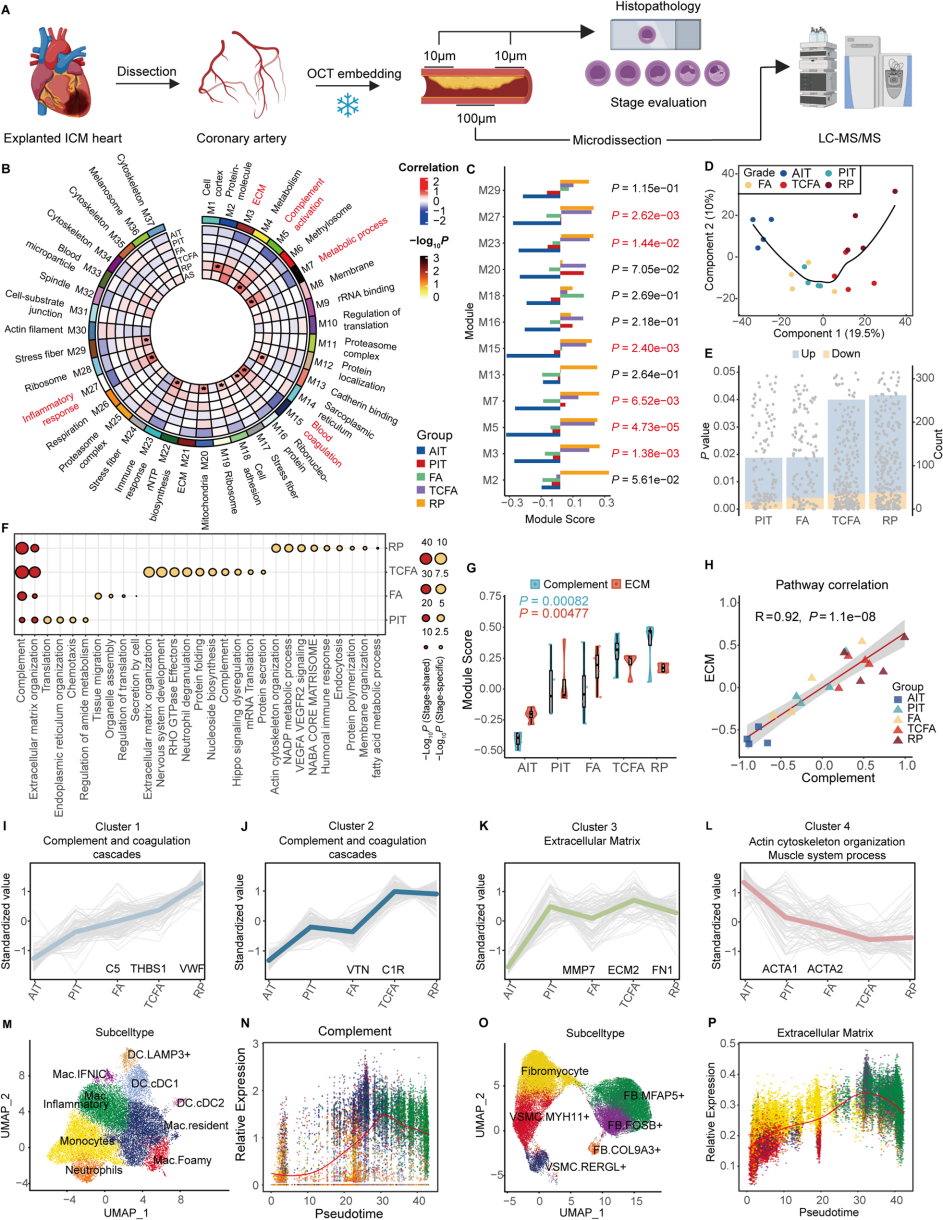
Proteomic and histopathologic profiling of different histopathologic stages of atherosclerosis. (**A**) Workflow of sample acquirement for histopathologic and proteomic analysis in the study. 20 human coronary segments across different pathological stages from explanted hearts of 4 ischemic cardiomyopathy patients were included for proteomic analysis. (**B**) A protein co-expression network for 2,229 proteins was built using weighted correlation network analysis (WGCNA), which consisted of 37 protein co-expression modules. Module eigenproteins were correlated with atherosclerosis, AIT, PIT, FA, TCFA, and RP. The cell type nature of each module was assessed by module protein overlap with cell-type-specific marker lists of endothelial cell (EC), fibroblast (FB), macrophage (Mac), and smooth muscle cell (SMC). Groups with *P* < 0.05 are marked with an asterisk (*), and the module names of the top 5 modules with a correlation coefficient (r-value) greater than 0.6 and ranked by significance are highlighted in red. AIT, adaptive intimal thickening; PIT, pathological intimal thickening; FA, fibroatheroma; TCFA, thin-cap fibroatheroma; RP, ruptured plaque. (**C**) Module eigenproteins score of the modules that are significantly associated with atherosclerosis. Module scores were grouped by those that change in different stages. Differences in module eigenprotein were assessed by one way ANOVA tests. *n* = 4 samples each group. Each spot represents one sample. *P* < 0.05 is marked in red. (**D**) Partial least-squares discriminant analysis (PLS-DA) of the proteome according to different stages. (**E**) Bar plot shows differential expression protein (DEP) numbers of PIT, FA, TCFA, and RP versus AIT, respectively. Each point represents a DEP, with the *P* value indicating the statistical significance of its expression change. The bars in the background show the count of DEPs in each category with the color denoting the direction of expression change: light blue for upregulated DEPs, and orange for downregulated DEPs. Upregulated DEPs with a fold change > 1.3, *P* <0.05 and downregulated DEPs with a fold change < 1/1.3, *P* < 0.05. Differences were assessed by student t-tests. (**F**) Gene ontology enrichment analysis of canonical and stage-specific pathways. (**G**) ssGSEA scores of complement and ECM organization across histopathologic stages. (**H**) Correlation analysis between complement and ECM organization pathway activities. The correlation was calculated using Pearson method. (**I**-**L**) K-means clustering of 397 DEPs revealing 4 clusters. Each line represents one protein. The line color indicates the degree of pattern correlation between each protein and the eigenvalue of the cluster. Hub proteins and enriched pathways are shown. (**M**) UMAP projection of myeloid sub-cell types. Mac.Fomay upregulated GPNMB, APOE, APOC1, characterized in lipid metabolism. Mac.Resident upregulated C1QA, C1QB, C1QC, LYVE1, characterized in tissue-resident. Mac.Inflammatory upregulated CCL2, CCL4L2, CXCL3, IL1B, characterized in inflammatory response and migration. Mac.IFNIC upregulated ISG15, IRF7, IFIT1, characterized in interferon-responsive. (**N**) Pseudotime analysis demonstrated complement pathway expression patterns along trajectories in myeloid cells. (**O**) UMAP projection of fibroblasts and VSMCs sub-cell types. MYH11⁺VSMCs upregulated MYH11, CNN1, characterized in actin and contractile function. RERGL⁺VSMCs upregulated RERGL, RGS16, FABP4, characterized in migration and fatty acid metabolism. Fibromyocytes upregulated LTBP2, FXYD5, TNFRSF11B, exhibit dual SMC contractile/myofibroblast properties, characterized in ECM remodeling. MFAP5⁺FB upregulated FBLN1, MFAP5, characterized in ECM secretion. c upregulated FOSB, MYC, KLF4, ATF3, characterized in stress response and proliferation. COL9A3⁺FB upregulated CYBP1, THBS4, ANGPTL7, characterized in vascular stabilization and angiogenesis. (**P**) Pseudotime analysis demonstrated ECM organization pathway expression patterns along trajectories in fibroblasts and VSMCs cells

To identify plasma biomarkers associated with clinical stage of coronary atherosclerosis, we performed plasma proteomics in a discovery cohort of 80 patients with angina undergoing coronary angiography (CAG). Subjects were stratified into CAG-negative (control) and coronary artery disease (CAD) groups [including acute coronary syndrome (ACS) and stable CAD patients] (Fig. 2A, Fig. S5A and Table S6). Through a stepwise approach, we selected 65 tissue and plasma overlapping complement and ECM proteins for subsequent biomarker selection [12] (Fig. 2B, Fig. S5B and Table S7). Least absolute shrinkage and selection operator regression (LASSO) identified THBS1, C1R, and ECM2 as representative biomarkers for identifying CAD (Fig. 2C and D). The trends of these biomarkers in plasma paralleled their expression dynamics in coronary tissue in different histopathologic stages (Fig. 2E-G, Fig. S5C-S5E). These three protein biomarkers were all positively correlated with the complexity and severity of CAD (Fig. 2H-J). The combination of three biomarkers discriminated CAD patients from the control group, with an area under the curve (AUC) of 0.831 (training set) and 0.764 (test set) (Fig. 2K). This panel also identified ACS from all angina individuals (AUC of 0.786) and distinguished stable CAD from the control group with AUC of 0.765 (Fig. 2L). In an external validation cohort (*n* = 113) (Table S8), plasma levels of THBS1, C1R, and ECM2 were significantly elevated in both stable CAD and ACS groups compared to controls (Fig. 2M-O). Receiver operating characteristic (ROC) analysis revealed moderate diagnostic performance for each biomarker in discriminating CAD, with AUCs of 0.701 (THBS1), 0.708 (ECM2), and 0.720 (C1R), respectively (Fig. 2P). Both univariate logistic analysis in different subgroups and multivariate logistic regression model confirmed the independent association between these biomarkers and CAD after adjusting for multiple clinical confounders (Table S9 and Table S10). The combined panel achieved good diagnostic efficiency, with AUCs of 0.869 and 0.822 for CAD and ACS detection respectively, and distinguished stable CAD from the control group with an AUC of 0.841 (Fig. 2Q). The diagnostic performance of the three-biomarker panel was consistently observed across clinical subgroups stratified by age, sex and other comorbidities, maintaining its significant predictive value for CAD (Fig. 2R). Furthermore, THBS1 and C1R outperformed high-sensitivity C reactive protein in identifying CAD (AUC: 0.697 and 0.764, respectively, Fig. S5F) and ACS (AUC: 0.691 and 0.716, respectively, Fig. S5G), while ECM2 displayed moderate performance in distinguishing CAD (AUC: 0.693) (Fig. S5F). Integrating the combined biomarker panel with clinical parameters resulted in a significant improvement in CAD diagnosis, suggesting its potential for clinical application (Fig. 2S, Fig. S5H and Table S11). Consistent with plasma findings, coronary immunohistochemistry revealed a concordant trend in THBS1, ECM2, and C1R expression across atherosclerosis progression (Figure S6).

**Fig. 2:**
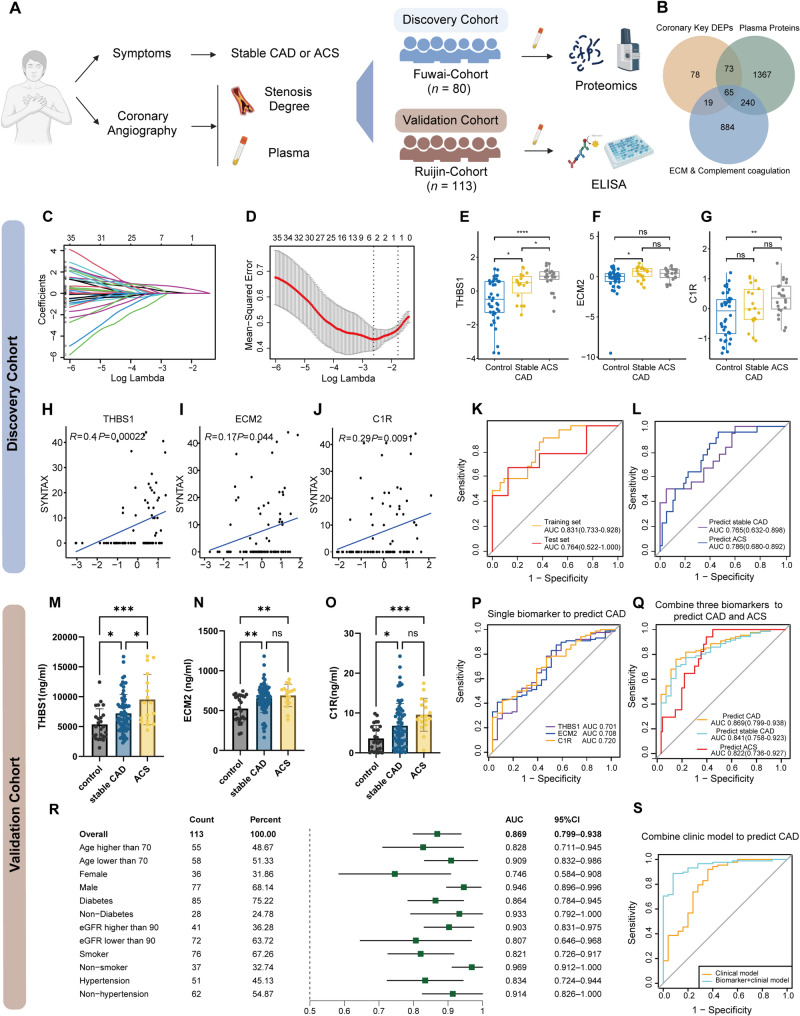
Clinical discovery and validation of biomarkers for coronary artery disease. (**A**) Study design, plasma samples and clinical information collection strategies of biomarker discovery and validation. The Discovery cohort [*n* = 80; 40 controls, 40 coronary artery disease (CAD)] underwent proteomic analysis to identify candidate biomarkers. Performance of biomarkers was subsequently validated using ELISA in an independent external validation cohort (*n* = 113; 25 healthy controls, 88 CAD patients). (**B**) Venn diagram of overlap strategy for coronary key DEPs, ECM organization and complement/coagulation proteins and plasma proteins. (**C**-**D**) Least absolute shrinkage and selection operator (LASSO) coefficient profiles of the plasma proteins feature for CAD and Lamda selection in the LASSO model used 5-fold cross-validation via minimum criteria. (**E**-**G**) Comparison of THBS1, ECM2, and C1R levels in Control, stable CAD, and ACS subgroups. *P*-values were adjusted using Bonferroni method. Protein levels were transformed using the z-score method. ns *P* > 0.05, * *P* < 0.05, **P < 0.01, ***P < 0.001, **** *P* < 0.0001. (**H**-**J**) Correlation of the THBS1, ECM2, and C1R levels with SYNergy between PCI with TAXUS and Cardiac Surgery (SYNTAX) score. Correlation coefficients were generated using the Spearman method. Protein levels were transformed using the log2 z-score method. (**K**) Receiver Operating Characteristic (ROC) curves for the logistic regression model incorporating THBS1, ECM2 and C1R in distinguishing CAD in training and test sets. (**L**) ROC curves for the model in distinguishing stable CAD from the control and identifying the ACS patients among all individuals in the discovery cohort.(**M**-**O**) Comparison of THBS1, ECM2, and C1R levels in control, stable CAD, and ACS subgroups. P-values were generated using ANOVA and adjusted using Tukey’s method. Protein levels were measured using ELISA method. Data are presented as mean ± SEM. (**P**) ROC curves for each single biomarker in distinguishing CAD in the validation cohort. (**Q**) ROC curves for the biomarkers-based model in distinguishing CAD, stable CAD and identifying the ACS patients in the external validation cohort. (**R**) Subgroup analysis of biomarker panel diagnostic performance. Forest plot displaying area under the curve (AUC) values with 95% confidence intervals for the biomarker panel across all analyzed subgroups. (**S**) ROC curves comparing diagnostic models. The combined biomarker-clinical model (AUC = 0.947, 95% CI: 0.906–0.988) shows superior sensitivity across specificity ranges compared to the clinical model alone (AUC = 0.82, 95% CI: 0.714–0.925). Variables in clinical model include age, sex, systolic blood pressure, diabetes, renal function, smoking, LDL-C, TG, Cystatin C levels and lipid lowering therapy. biomarker-clinical model includes variables in clinical model and three biomarkers

This study established histopathologic stage-resolved proteomic profiling of coronary atherosclerosis and demonstrated the translational potential of proteomics to identify clinically relevant biomarkers. Nevertheless, the study was limited by its small sample size of patient specimens. Future large-scale, multi-center prospective studies are warranted to validate the biomarker utility.

## Supplementary Information

Supplementary Material 1.

## Data Availability

The data underlying this study are available from the corresponding author upon reasonable request.
