# Supplementary material for: Proteomic and histopathologic profiling reveal molecular features and clinical biomarkers of coronary atherosclerosis
Source: Biomark Res. 2025 Oct 22;13:132. doi: 10.1186/s40364-025-00846-3 (PMC12548267; doi:10.1186/s40364-025-00846-3)
Supplement: Supplementary file 1 — Supplementary Material 1. [file 40364_2025_846_MOESM1_ESM.docx]

**Proteomic and histopathologic profiling reveal molecular features and clinical biomarkers of coronary atherosclerosis:** Supplementary Materials

**Supplementary Methods**

The data not provided in this article that support the findings of this study are available from the corresponding authors upon reasonable request.

**Study population**

In the tissue proteomics analysis, the coronary artery specimens were collected from the explanted hearts of 4 patients with ischemic cardiomyopathy from the Heart Transplantation Center of Fuwai Hospital, China. The angiography was performed and reviewed to evaluate the segments of stenosis and thrombosis before each transplantation surgery.

For plasma proteomics validation and biomarkers panel discovery, a discovery cohort of 80 patients with angina who underwent coronary angiography (CAG) for the first time between September 2020 to January 2021 were collected from Fuwai hospital. The exclusion criteria included history of coronary artery bypass grafting, percutaneous coronary intervention within the past 6 months, chronic heart failure with New York Heart Association class III or IV, pulmonary heart disease, renal failure requiring hemodialysis, immune system disorders, or malignant tumor. The participants included 40 CAG-negative (control) patients and 40 age-matched CAD patients, among whom 22 patients presented with acute coronary syndrome (ACS) and 18 patients had stable coronary stenosis (stable CAD).

For ELISA validation of the identified plasma panel, with the same inclusion and exclusion criteria, another cohort of 113 patients who underwent CAG for the first time was collected from Ruijin Hospital, as the validation cohort.

Levels of glucose, triglyceride, total cholesterol, low-density lipoprotein cholesterol, and high-density lipoprotein cholesterol, were assessed using a standard laboratory protocol (HITACHI 912 Analyzer; Roche Diagnostics, Germany). Glomerular filtration rate (GFR) was estimated using the Chronic Kidney Disease Epidemiology Collaboration equation.

This study followed the ethical standards of the Declaration of Helsinki. The protocol was approved by the Ethics Committee of Fuwai Hospital, Chinese Academy of Medical Sciences and Peking Union Medical University, as well as by the Ministry of Science and Technology, with the approval number of FW- 2024-2480. All patients provided written informed consent for the research use of the samples.

**Coronary artery sample collection**

For processing coronary artery samples, we immediately perfused the whole explanted hearts with histidine-tryptophan-ketoglutarate (HTK) cardioplegia, followed by dissection on ice. The main branches of coronary arteries were completely isolated from the heart including the left main coronary artery (LMCA), left anterior descending (LAD), left circumflex branch (LCX), and right coronary artery (RCA). The fat and connective tissues around the vessels were removed carefully. The arteries were further dissected into 0.5 cm segments and embedded by optimal cutting temperature compound (OCT), followed by frozen at -80℃.

Specimens were cut into a series of sections in an order of 10 µm - 100µm - 10 µm (Leica RM2265, Germany). The 10 µm thickness sections were used for hematoxylin and eosin (H&E) staining subsequently (defined as histopathologic section), while the 100µm sections were used for proteomics (defined as proteomic section). The histopathology of coronary sections was categorized as AIT, PIT, FA, TCFA, and RP stages of atherosclerosis by H&E staining according to the modified AHA Classification^1^. The plaque erosion was not included in this study. The histopathology of the 100µm thickness segment was defined according to the two 10µm sections next to this segment. When the histopathologic diagnosis of two adjacent histopathologic sections was inconsistent, the proteomic section in the middle of the two sections was excluded for subsequent study. In each explanted heart, we obtained the sections covering five histopathology stages. The proteomic sections were pooled together according to their categories; and five samples were obtained for further proteomic profiling analysis in each heart.

**Coronary artery sample preparation for proteomics**

Quantitative proteomics based on liquid chromatography-tandem mass spectrometry (LC-MS/MS) was applied to detect the comprehensive protein map prepared from the coronary vessel samples under different disease stages. The pooled vessel tissue was washed twice with PBS to remove OCT and homogenized in 8 mol/L urea in PBS. The tissue extracts were then centrifuged at 14,000 g for 30 minutes at 4℃. The BCA protein assay kit (SolarBio, Beijing) was used to determine protein concentrations. Extracted 200 μg proteins were reduced with 5 mM dithiothreitol (DTT) and alkylated with 12.5 mM iodoacetamide (IAM). The protein solutions were diluted to 1.5 M urea with PBS, followed by digestion using sequencing grade modified trypsin (Promega, Fitchburg, WI) of a 1:50 protease/protein ratio at 37℃ overnight.

To quantitate the expressed proteins in each sample, tandem-mass-tagging reagent (TMT, Thermo, Pierce Biotechnology, Rockford, IL) was applied to each atherosclerotic sample, and the unaffected sample as the normal standard.^2^ The samples were desalted using Sep-Pak C18 Vac cartridges (Waters, Milford, MA). The extracts were centrifuged in SpeedVac to dry them completely. The purified peptides were resuspended in 100 μL of 100 mM triethyl ammonium bicarbonate (TEAB). Next, 20 μL of TMT labeling reagent was added to each peptide solution and allowed to react for one hour.

The 5 samples (AIT, PIT, FA, TCFA, RP samples) from each heart were put in a single run, and thus we performed 4 runs for 4 explanted hearts. Peptides from samples with different stages were labeled using TMT6-126 (AIT), TMT6-127 (PIT), TMT6-128 (FA), TMT6-129 (TCFA), and TMT6-130 (RP) reagents (TMT, Thermo, Pierce Biotechnology, Rockford, IL). Next, 5% hydroxylamine was used to quench the reactions for 15 minutes. The TMT-labeled peptides were mixed and desalted using Sep-Pak C18 Vac cartridges. The isobaric tag-labeled peptides were then fractioned using HPLC and sent to LC-MS/MS for analysis.^16^

The peptide mixture was fractioned by a UPLC 3000 system (Thermo-Fisher Scientific, Waltham, MA) with an XBridge C18 RP column (5 μm, 150 Å, 250 mm × 4.6 mm i.d., (Waters, Milford, MA)). Mobile phase A consisted of 98% H2O and 2% acetonitrile (pH 10.0). Mobile phase B consisted of 98% acetonitrile and 2% H2O (pH 10.0). Ammonium hydroxide was added to raise the pH to 10. Peptides were separated with the following gradients: 5% to 8% B, 5 min; 8% to 18% B, 25 min; 18%-32% B, 32 min; 32%-95% B 6min; 95%-5% B, 5 min. Peptide peaks were detected by 280 nm absorbance, 48 fractions were collected, combined into 12 fractions after drying by speed vac, and then resolved in 0.1% formic acid.

**LC-MS/MS analysis of coronary artery sample**

For LC-MS/MS analysis, the TMT-labeled peptides were separated by a 120-minute gradient elution at a flow rate of 0.250 μL/min with a Thermo-Dionex Ultimate 3000 HPLC system. The analytical column was a homemade fused silica capillary column (75 μm i.d., 150 mm length; Upchurch, Oak Harbor, WA) packed with C18 resin (300Å, 5 μm; Varian, Lexington, MA). Mobile phase A consisted of 0.1% formic acid and mobile phase B consisted of 100% acetonitrile and 0.1% formic acid. A Q-Extractive mass spectrometer was operated in the data-dependent acquisition mode using Xcalibur 3.0 software and there was a full-scan mass spectrum in the Orbitrap (300-1800 m/z, 70, 000 resolution) followed by 20 data-dependent MS/MS scans at 32% normalized collision energy (HCD). The mass window for precursor ion selection was 2.4 m/z, the threshold for triggering MS/MS experiments was 1.7×10^4^, and the dynamic exclusion time was 20 s.

**Proteomics data processing and bioinformatics analysis**

The MS/MS spectra from each LC-MS/MS run were analyzed as previously reported.^3^ Briefly, the original data was searched against the UniProt human database (version January 10, 2015; 89105 sequences) using the SEQUEST searching engine of Proteome Discoverer software (version 2.1) from Thermo-Fisher Scientific. The search criteria included: 1) full tryptic specificity was required; 2) two missed cleavage was allowed; 3) the oxidation (M) was set as the variable modification; 4) carbamidomethylation (C) and TMT six plex (N-terminal and K) were set as the fixed modifications; 5) precursor ion mass tolerance was set at 20 ppm for all MS acquired in an Orbitrap mass analyzer; and 6) the fragment ion mass tolerance was set at 20 mm for all MS2 spectra acquired. An identified peptide with a confidence value of high was considered as identification and peptide spectral matches (PSMs) were validated. The peptide false discovery rate (FDR) was estimated using the percolator function provided by PD, and the cutoff score was accepted at 1% based on the decoy database. The protein quantification was performed using PD software according to the manufacturer's instructions on the intensity of six TMT reporter ions per peptide. Protein abundance was calculated as the median of all peptide hits belonging to a protein. Quantitative precision was expressed as protein ratio variability. Significant differentially expressed proteins were determined by a two-tailed Student's t-test. And p-value of less than 0.05 and a fold change ratio of greater than 1.3 was considered significant.

To perform the pathway enrichment analysis, Metascape (http://www.metascape.org/)^4^ was used. The canonical pathways, overrepresented biological functions, and molecular networks were generated based on the Ingenuity Knowledge Base and Gene Ontology (http://www.geneontology.org/).

**Plasma sample preparation for proteomics**

Blood samples were obtained from all patients on the day of angiography after overnight fasting and were stored at −80 °C before analysis. The samples were centrifuged at the freezing temperature at 1500g for 15 minutes. Supernatant plasma was stored at −80°C before analysis.

The n-LAPE/MS TM Kit was purchased from ProteinT Biotechnology Co., Ltd.. 40 µL plasma samples were diluted with 10 µL pre-mixed beads and 260 µL Binding Buffer. The samples were incubated at 37 °C for 10 min with shaking at 1,000 rpm. After incubation, the tubes were put on a magnetic frame for 2 min for separation. Unbound proteins in the supernatant were pipetted out. The enriched beads were further washed with 500 µL Washing Buffer three times with magnetic separation.

The beads were resuspended with 50 µL Lysis Buffer containing TCEP and CAA and heated at 95 °C for 10 min with shaking. After cooling to room temperature, 2 µL trypsin digestion buffer was added, and incubated at 37 °C for overnight with shaking. 150 µL Desorption Buffer was added and the supernatant was processed with the SDB column method for desalting. The eluted peptides were dried and dissolved in a 20 µL Loading Buffer containing iRT. The peptide concentration was measured by Nanodrop for MS detection.

**DIA of LC-MS/MS**

Equal amounts of iRT (purchased from Biognosys) were added into each DDA or DIA run on a Thermo Scientific U3000 nanoflow LC system followed by a Q Exactive HF mass spectrometer. Peptide samples were dissolved with loading buffer (2% ACN) and separated on a 150 μm ID × 30 cm column (C18, 1.9 μm, 120 Å, Dr. Maisch GmbH) using a 150 min gradient (A: 2% ACN, 0.1% FA; B: 80% ACN, 0.1% FA; 0-5 min, 3-6%B; 6-44 min, 6-90%B; 45-54 min, 90%B; 55-60 min, 6%B) with a flow rate of 600 L/min. The spray voltage was set at 2,000 V in positive ion mode, and the ion transfer tube temperature was set at 270. For DIA a 60 K resolution MS scan @ m/z 200 MS was performed, and the AGC target value was set at 1e6 or 20 ms of max injection time by the orbitrap mass analyzer (350–1,500 m/z). The MS/MS AGC target value was set at 1e6 with an auto setting of max injection time generated by HCD fragmentation at a resolution of 30,000 @ m/z 200. The NCE was set at NCE 28%. For proteome DIA MS runs, fragment analysis was subdivided into 40 DIA isolation windows with different widths depending on the DDA search results. MS scans were also performed before each DIA cycle.

**Plasma Proteomics Data Processing and Bioinformatics Analysis**

The DIA data were searched against the human UniProt database (20,365 sequences) using DIA-NN (v1.8.1) with default settings, with trypsin/P digest rule, high protein and peptide confidential level, and FDR of 0.01. The proteomics dataset was processed as follows: (1) Protein filtering such that proteins with at least 75% of valid values were kept; (2) Data normalization with log2 transformation and mean centering of the dataset. Imputation of missing values was performed using K-Nearest Neighbor approach (KNN).

**Weighted Gene Co-Expression Network Analysis**

The WGCNA package in R was used to identify distinct protein modules among the 2,229 proteins used for analysis^5^. A weighted power adjacency matrix was constructed using unsigned correlations between proteins and mapping 6 the results onto the -1-1 interval. The power parameter was selected such that the topological overlap connectivity (k) of the entire network approximated a scale-free topology. The topological overlap was used to create a dissimilarity matrix for hierarchical clustering to identify modules.

**K-means clustering**

K-means clustering was performed using the R package ‘stats’ (v4.4.2) after log2-transformation and Z-score scaling of the data. We calculated the minimum centroid distance for a range of cluster numbers and the optimal number was chosen using the ‘elbow’ method.

**Single sample Gene Set Enrichment Analysis (ssGSEA) analysis**

Single sample gene set enrichment analysis (ssGSEA) was implemented via the gsva function from the GSVA package (https://github.com/rcastelo/GSVA), explicitly specifying method = "ssgsea" to activate the rank-based enrichment scoring algorithm.

**Single-cell data integration and cell type annotations**

The data matrices from different studies were merged and log-normalized (size factor 10,000) using the python module scanpy (Version 1.10.4). The top 2000 highly variable genes using scanpy.pp.highly_variable_genes function for downstream analysis. We then scaled the expression data with the function scanpy.pp.scale and performed principal component analysis (PCA) with scanpy.tl.pca function. The first 30 principal components (PCs) were selected for batch effect correction via scanpy.external.pp.harmony_integrate function for batch variable SampleID. Then we used the scanpy.pp.neighbors function with parameter n_neighbors =30 and n_pcs=30 to construct a k-nearest neighbor graph with above 30 corrected PCs. Finally, we used the scanpy.tl.leiden function to cluster the cells with resolutions from 0.1 to 1.0, and a resolution of 0.4was selected for the final clustering.

A two-dimensional Uniform Manifold Approximation and Projection (UMAP) was applied to visualize cell clusters via the scanpy.tl.umap function. Cell types were identified mainly based on expression patterns of canonical markers for major cell types and differentially expressed genes for each cluster. To identify subpopulations within the endothelial cell (EC), Macrophage cell, Fibroblast/VSMC and NK/T, scanpy.pp. neighbors, scanpy.tl.umap and scanpy.tl.louvain functions were performed again on the corrected harmony embedding.

**Trajectory analysis**

Trajectory analysis based on monocle2 of selected clusters was performed with DDRTree dimensionality reduction and the order_cells function of the Monocle2 framework (https://github.com/MaxMeieran/monocle2). The top genes obtained for each cluster were ranked using the differentialGeneTest function, and trajectories were visualized through plot_cell_trajectory and plot_pseudotime_heatmap workflows to infer cellular differentiation dynamics across pseudotime-ordering branches.

**ELISA assay**

The diagnostic accuracy of THBS1, C1R and ECM2 were validated in the plasma of the external cohort by ELISA assay. ELISA kits used of THBS1, C1R and ECM2 were as follows, Elabscience #E-EL-H1589, EIAab #E16460h, EIAab # E11797h, respectively. The concentrations of these biomarkers in plasma were determined according to the manufacturer’s protocols. Absorbance was measured using a microplate reader (infinite-M200, Tecan). All samples were analyzed in triplicate, and the average concentration for each patient was calculated.

**Biomarker selection and modeling**

To identify the key blood protein that can distinguish the CAD from CAG-negative control group, the discovery patient’s cohort was randomly divided into training and validation sets (7: 3). For biomarker selection, least absolute shrinkage and selection operator (LASSO) logistic regression using the “glmnet” R package within the training data, fivefold cross-validation was implemented to choose the key protein candidates out of the 65 biomarkers. A logistic regression model for predicting positive coronary angiography (CAG) results was constructed using the selected proteins in the training set and validated in the test set. The model’s generalizability was further evaluated by distinguishing: 1) stable CAD from controls; 2) acute coronary syndrome (ACS) patients from non-ACS individuals. For external validation, the predictive performance of individual and combined biomarkers for identifying positive CAG were also analyzed. The area under the receiver operating characteristic (ROC) curve (AUC) was used as a measure of the predictive ability of the protein-based model.

**Immunohistochemistry**

The human coronary artery tissues were sectioned into 4 μm-thick sections. Then, the tissue sections were dewaxed in xylene and rehydrated using a graded series of ethanol. After dewaxing and rehydration, the sections were washed with PBS, and 3% H2O2 was used to deplete endogenous peroxide. Next, sections were heated in a water bath in Ethylene Diamine Tetraacetic Acid (EDTA) buffer at 95 ℃ for 15 min and then cooled for 15 min at room temperature. After that, goat serum was added to the tissues to block nonspecific adsorption sites at 37 ℃ for 30 min. Subsequently, these tissue sections were incubated overnight with primary antibodies (THBS1 antibody: abcam, # ab267388; C1R antibody: invitrogen, # PA5-28028; ECM2 antibody invitrogen, # PA5-144760) at 4 ℃. The next day, the sections were washed extensively with PBS, and HRP-conjugated secondary antibody was added for 20 min at 37 ℃ After the sections were rinsed, DAB solution was applied and incubated for 5 min at room temperature, and sections were counterstained with hematoxylin. Digital images were acquired on Pannoramic 250 FLASH Ⅲ (3DHISTECH).

**Statistical analysis**

Continuous variables were summarized as mean ± standard deviation if normally distributed or median (interquartile range, IQR) for non-normally distributed data. Parametric variables (normally distributed) were compared using Student’s t-test (two groups) or one-way ANOVA (>two groups), while non-parametric variables (non-Gaussian distribution) were analyzed with Mann-Whitney U test (two groups) or Kruskal-Wallis H test (>two groups). Bonferroni correction was conducted for multiple comparsion adjustment in differential expressional analysis during the discovery stage. Post-hoc multiple comparisons were adjusted using Tukey’s method (ANOVA) or Dunn’s correction (Kruskal-Wallis). The biomarker selection and modeling can be found in the “Supplementary methods-Biomarker selection and modeling”. All statistical analysis were two-tailed, with P < 0.05 considered statistically significant. R (version 4.3.1) was used for the statistical analysis.

**Supplemental Figures and Legends**


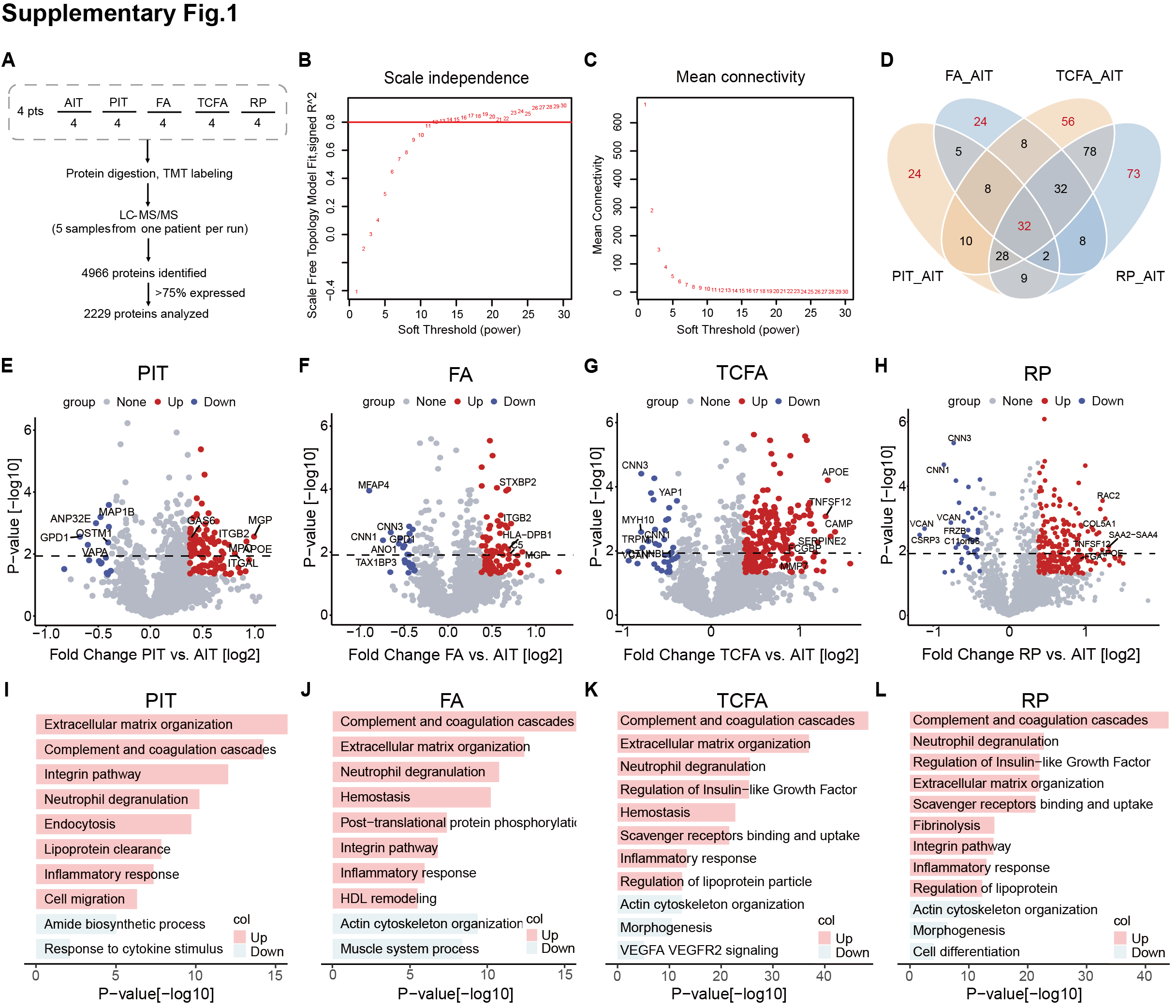


**Fig. S1 Power and topology scale of weighted co-expression network analysis and differential expression analysis**

(A)Schematic overview of the workflow for coronary artery proteomic profiling. 2,229 high-confidence proteins were quantified after filtering.

(B)Soft thresholding power selection in WGCNA analysis. Scale-free topology is best approximated when the adjacency power parameter β=14.

(C)Mean connectivity analysis of WGCNA.

(D)Veen plot shows stage-specific and stage-shared DEP numbers.

1. H) Volcano plot depicting DEPs between PIT and AIT(E), FA and AIT(F), TCFA and AIT(G), RP and AIT(H). Groups were compared by Student’s *t* test, P < 0.05 was considered significance. Broken lines in volcano plots represent a further Bonferroni corrected P-value significance level.
2. L) Bar plot showing enrichment pathways of DEPs between PIT and AIT(I), FA and AIT(J), TCFA and AIT(K), RP and AIT(L).


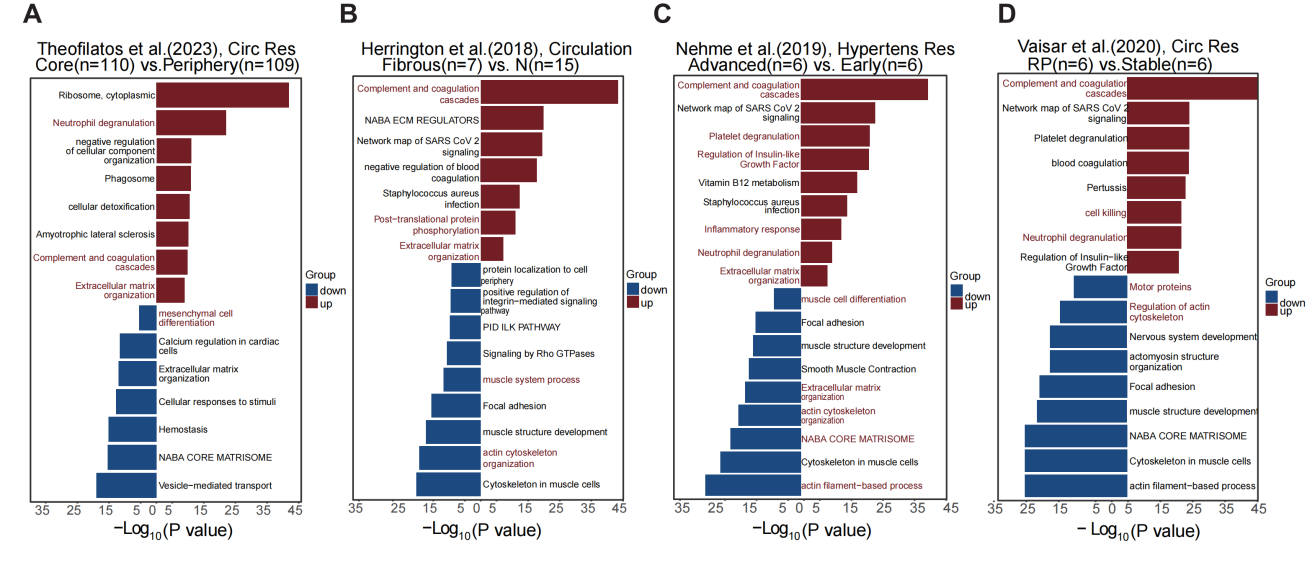


**Figure S2 Pathway enrichment of DEPs from reported human AS proteomic datasets**

(A) Pathway enrichment of DEPs of Theofiatos et al^6^. Core (*n* = 110) vs Periphery (*n* = 109); (B) Pathway enrichment of DEPs of Herrington et al^7^. Fibrous (*n* = 7) vs N (*n* = 5); (C) Pathway enrichment of DEPs of Nehme et al^8^. Advanced (*n* = 6) vs Early (*n* = 6); (D) Pathway enrichment of DEPs of Vaisar et al^9^., RP (*n*=6) vs Stable (*n*=6). The pathways overlapping with our DEPs enrichment are marked in red; the complement and ECM pathways are enriched in all datasets.


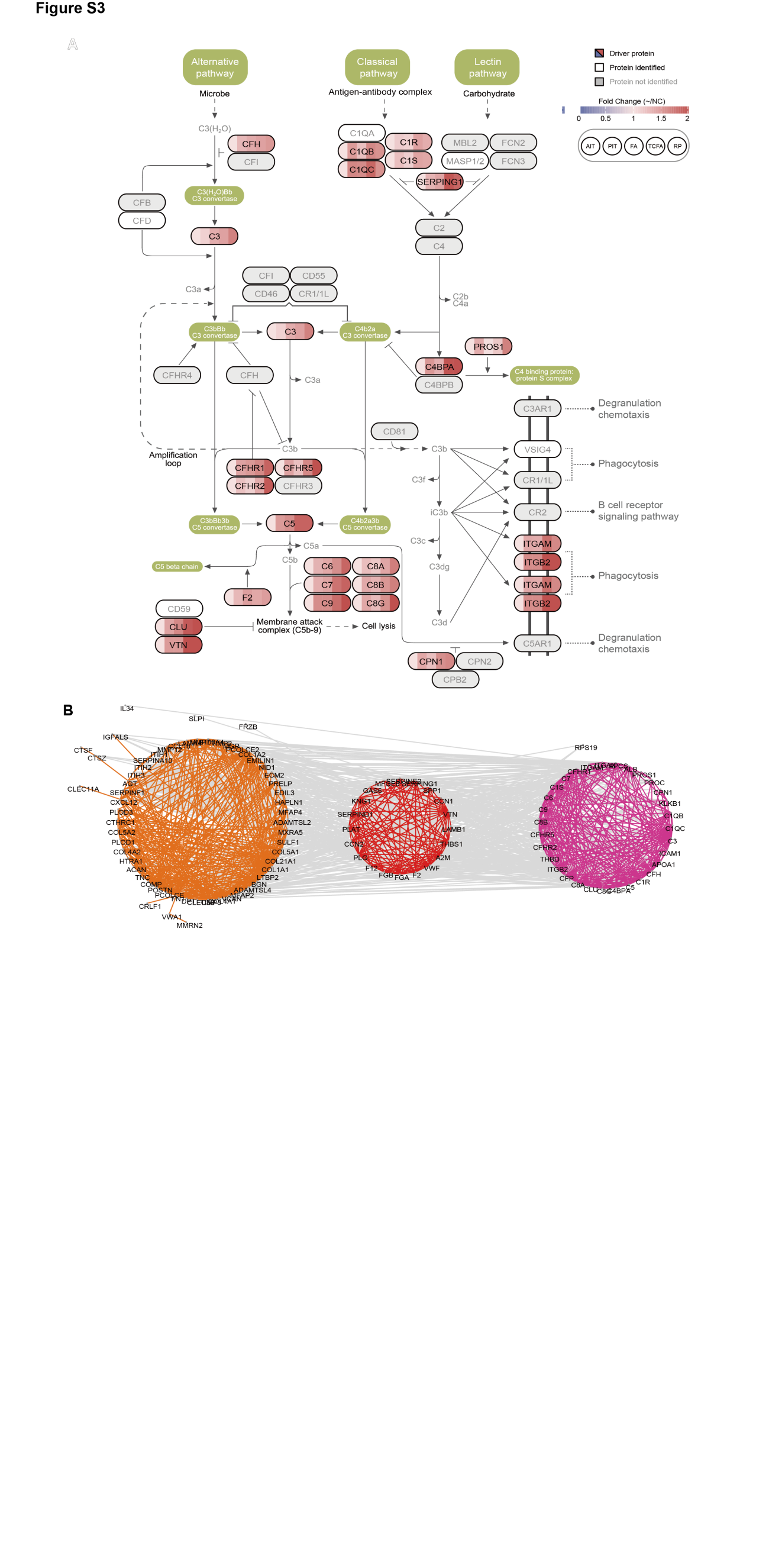


**Figure S3 Overview of complement pathways protein abundance**

Overview of complement pathways protein abundance across atherosclerosis stages (PIT, FA, TCFA, RP) compared to AIT. Color scale: red (upregulation), blue (downregulation).


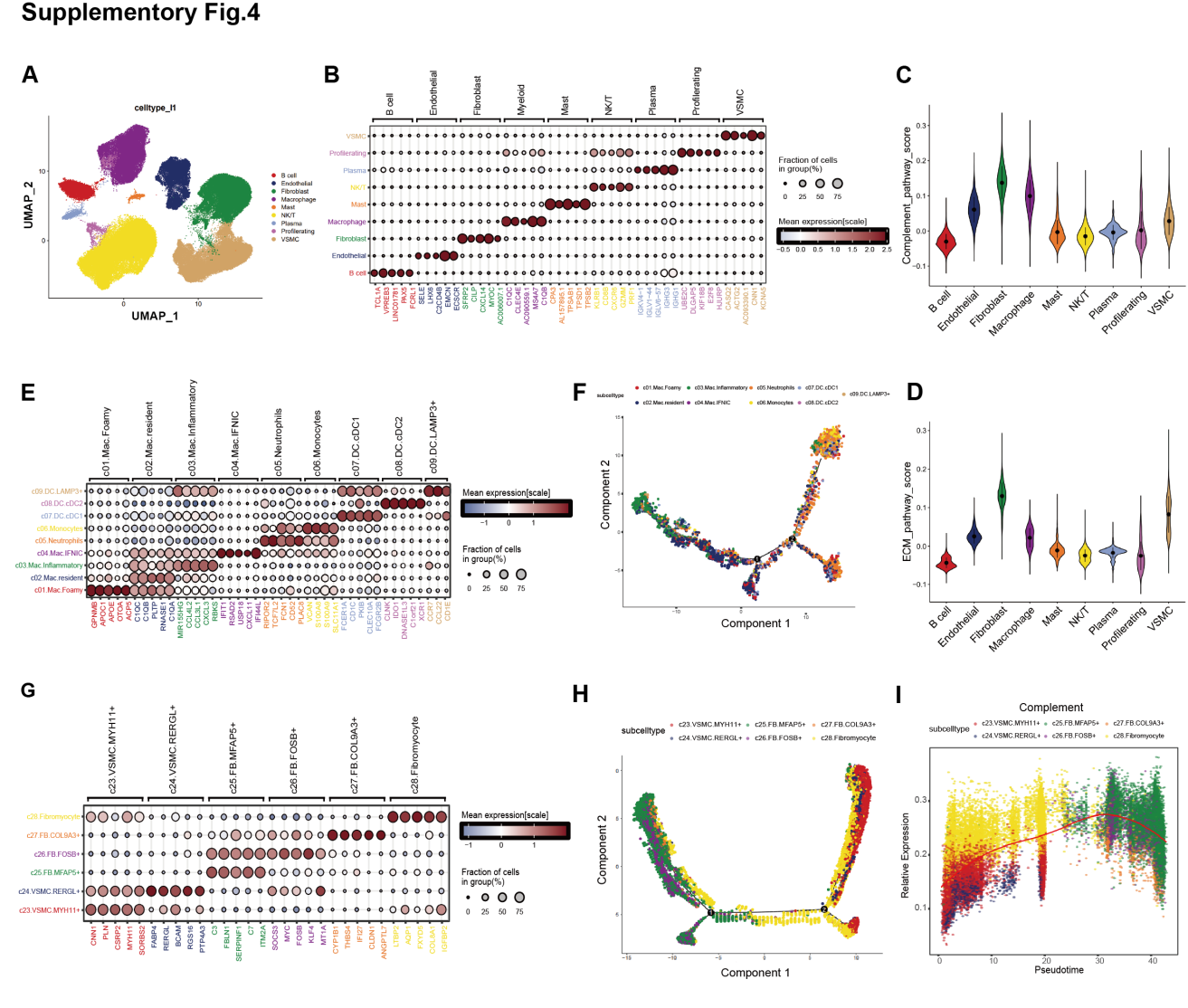


**Figure S4 Integration of the human coronary atherosclerosis single-cell RNA-seq data**

1. UMAP projection of a total of 142,729 cells from 38 samples of human coronary AS. Cell identity was annotated based on cell type-specific markers.
2. Dot plot of cell type marker expression. The dot plot shows the expression of marker genes for various cell types. Each dot represents the fraction of cells expressing the marker (dot size) and the mean expression level (color intensity).
3. Complement pathway score according to different cell types.
4. ECM pathway score according to different cell types.
5. Dot plot of myeloid sub celltype marker expression.
6. Pseudotime analysis of myeloid cells. The trajectories of myeloid cells originated from monocyte and neutrophil, and divided to 1) Mac.Resident and Mac.Foamy; 2) Mac.Resident and Mac.Inflammatory.
7. Dot plot of FBs and VSMCs subtype marker expression.
8. Pseudotime analysis of FBs and VSMCs. The trajectories of FBs and VSMCs originated from MYH11⁺VSMCs and RERGL⁺VSMCs and transited to Fibromyocytes and finally divided to MFAP5⁺FB, MFAP5⁺FB and COL9A3⁺FB.
9. Pseudotime analysis demonstrated complement pathway expression patterns along trajectories in myeloid cells.


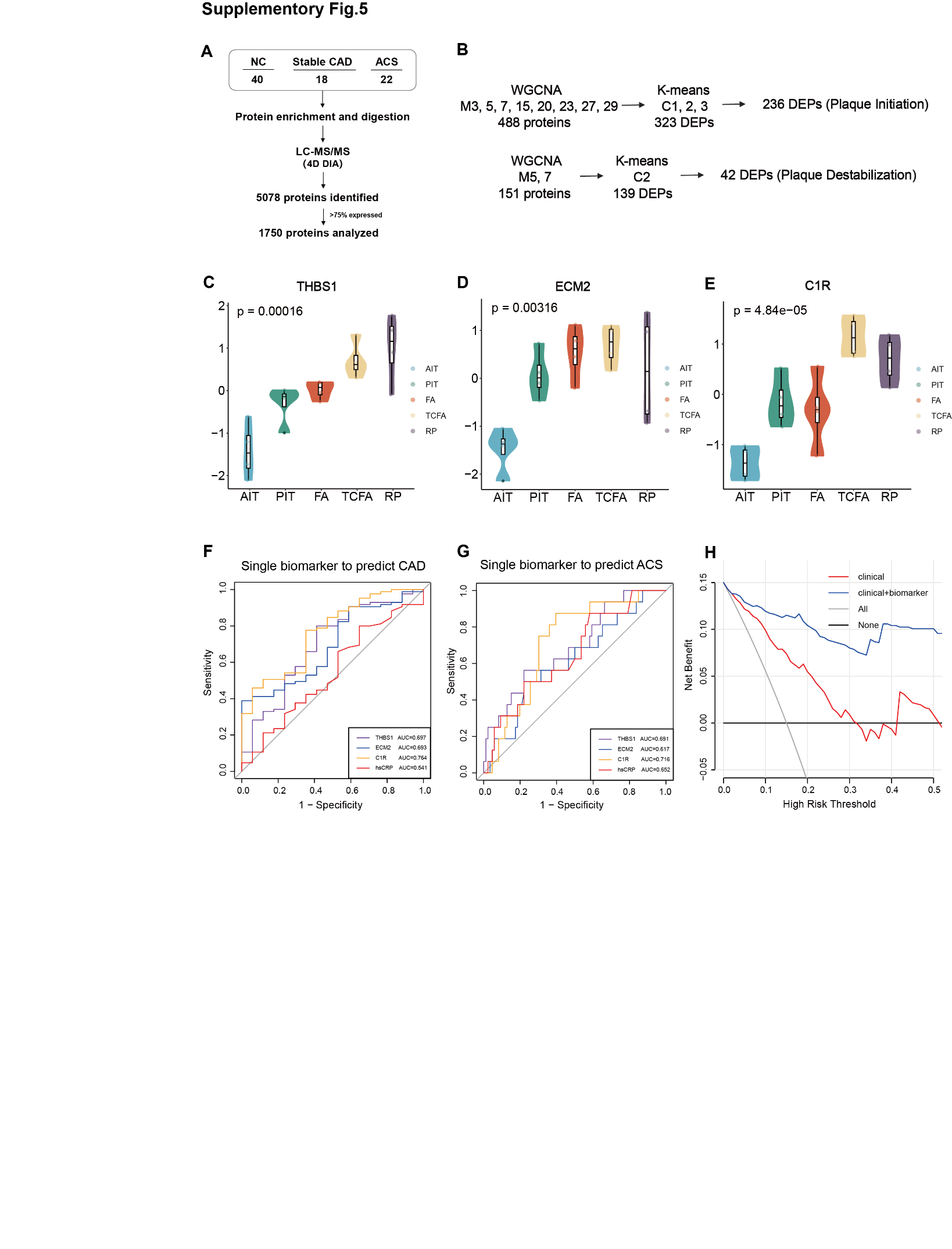


**Figure S5 Clinical association and plasma expression level of THBS1, C1R, ECM2**

(A)Workflow of the proteomic testing, protein quantification and filtering strategies. 1750 proteins were quantified after filtering. Adaptive intimal thickening (AIT) group, n = 40 samples. Stable coronary artery disease (CAD) group, n = 18 samples. Acute coronary syndrome (ACS) group, n = 22 samples.

(B) The process of determining coronary key DEPs (plaque initiation DEPs and plaque destabilization DEPs). Plaque initiation DEPs include proteins that are elevated from AIT to PIT, corresponding to WGCNA modules 3, 5, 7, 15, 20, 23, 27, and 29 as well as K-means clusters 1, 2, and 3, resulting in a total of 236 DEPs. Plaque destabilization DEPs include proteins that are significantly elevated from FA to TCFA, corresponding to WGCNA modules 5 and 7 as well as K-means cluster 2, resulting in a total of 42 DEPs. Together, these two categories comprise a total of 236 DEPs.

(C-E) Comparison of THBS1, ECM2, and C1R levels in the different stages of AS progress (AIT, PIT, FA, TCFA, RP) presented by human AS tissue samples. Protein levels were transformed using the z-score method.

1. Receiver operating characteristic (ROC) curves for single biomarkers (THBS1, ECM2, C1R) and hsCRP in predicting CAD (analysis was conducted in 102 patients with available hsCRP levels from the external validation cohort)
2. ROC curves for single biomarkers (THBS1, ECM2, C1R) and hsCRP in predicting ACS (analysis was conducted in 102 patients with available hsCRP levels from the external validation cohort)
3. Decision curve analysis (DCA) for CAD prediction when comparing clinical model and clinical + biomarker model. Clinical utility comparison of prediction models across risk thresholds (0-50%). Net benefit of the combined biomarker + clinical model (blue line) exceeds both the clinical model alone (red line) and reference strategies (treat all [grey], treat none [black]). Variables in clinical model include age, sex, systolic blood pressure, diabetes, renal function, smoking, LDL-C, TG, Cystatin C and lipid lowering therapy. Variables in clinical +biomarker model include the variables in clinical model as well as THBS1, C1R and ECM2.


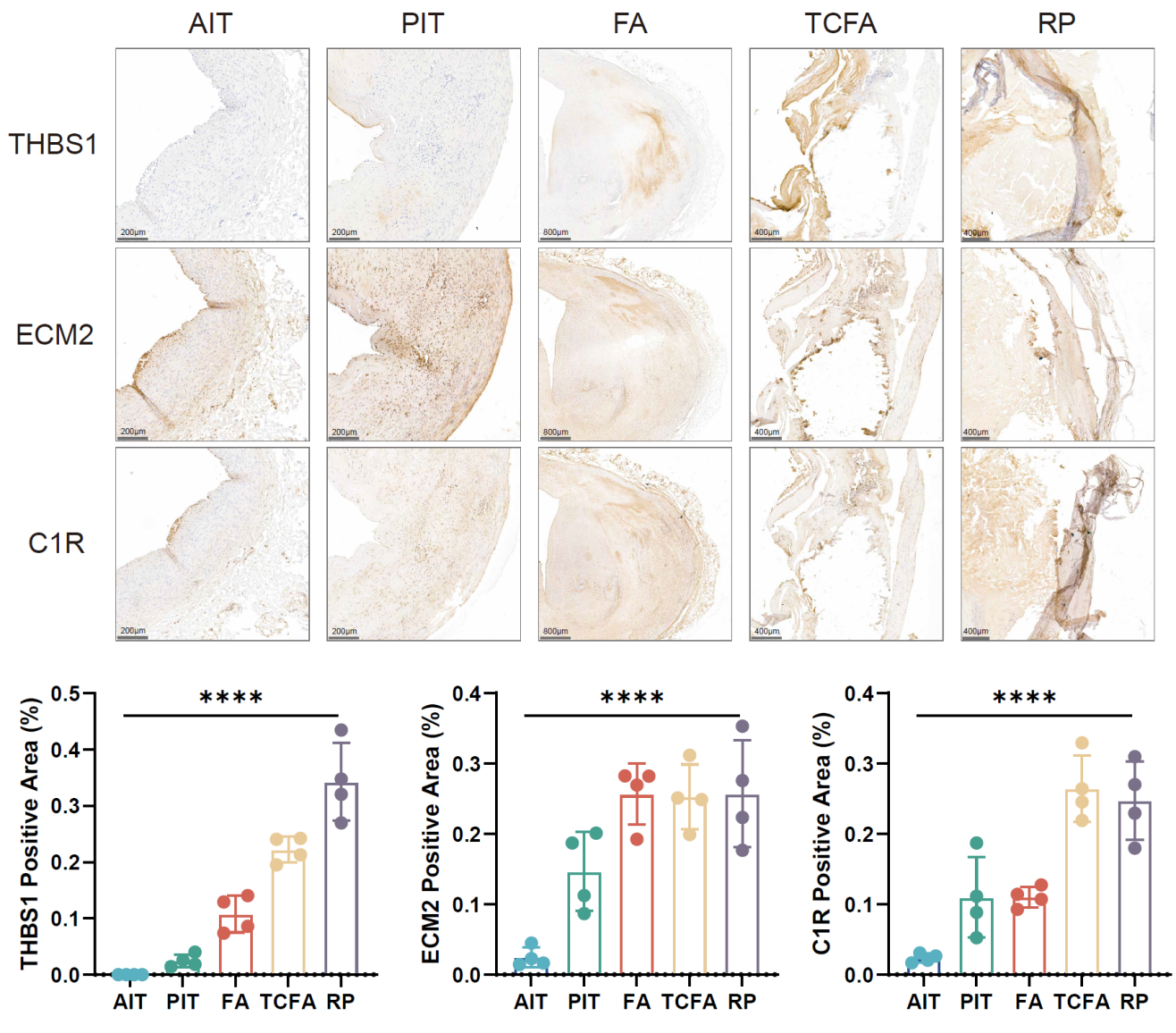


**Figure S6** Immunochemistry of THBS1, ECM2, C1R in different histopathologic stages of coronary artery. THBS1 exhibited continuously elevation from AIT, PIT, FA to TCFA, RP, corresponding to expression in proteomic findings. THBS1 specifically expressed in intima, fibrotic cap and lipid pool. ECM2 elevated from AIT to FA and remain stable, corresponding to expression in proteomic findings. ECM2 expressed widely in whole vasculature. C1R elevated in PIT and FA, and further elevated in TCFA. C1R also expressed widely in whole vasculature. Differences in histopathological stages were assessed by one way ANOVA tests. n = 4 samples each group. Each spot represents one sample. Data are presented as mean±SEM. **** P<0.0001.

**Supplementary Tables S1-11**

Table S1. Clinical characteristics of the patients undergone heart transplantation

| **ID** | **Race** | **Age** | **Sex** | **BMI** | **Etiology** | **LVEF** | **History of CABG** | **Antiplatelet** | **Statin** | **Beta-blocker** | **ACEI/**  **ARB** | **Spironolactone** |
| --- | --- | --- | --- | --- | --- | --- | --- | --- | --- | --- | --- | --- |
| HS0228 | Asian | 59 | Male | 22.46 | ICM | 14 | No | 1 | 1 | 1 | 1 | 1 |
| HS0230 | Asian | 64 | Male | 23.18 | ICM | 21 | No | 1 | 1 | 1 | 1 | 1 |
| HS0233 | Asian | 53 | Male | 20.76 | ICM | 30 | No | 1 | 1 | 1 | 1 | 1 |
| HS0253 | Asian | 47 | Male | 19.94 | ICM | 30 | No | 1 | 1 | 1 | 1 | 1 |
| BMI, body mass index; LVEF, left ventricular ejection fraction; CABG, coronary artery bypass graft; ACEI, angiotensin-converting enzyme inhibitor; ARB, angiotensin receptor blocker. | | | | | | | | | | | | |

Table S2. Weighted gene co-expression network analysis modules

|  | **AIT** | | **PIT** | | **FA** | | **TCFA** | | **RP** | | **AS** | |
| --- | --- | --- | --- | --- | --- | --- | --- | --- | --- | --- | --- | --- |
|  | **Correlation** | **P-value** | **Correlation** | **P-value** | **Correlation** | **P-value** | **Correlation** | **P-value** | **Correlation** | **P-value** | **Correlation** | **P-value** |
| M1 | -0.059 | 0.351 | 0.026 | 0.861 | -0.093 | 0.478 | 0.055 | 0.723 | 0.071 | 0.135 | 0.059 | 0.351 |
| M2 | -0.805 | 0.295 | -0.060 | 0.637 | -0.101 | 0.318 | 0.459 | 0.828 | 0.507 | 0.002 | 0.805 | 0.295 |
| M3 | -0.013 | 0.004 | -0.044 | 0.685 | -0.029 | 0.393 | 0.019 | 0.093 | 0.067 | 0.018 | 0.013 | 0.004 |
| M4 | -0.032 | 0.628 | 0.005 | 0.811 | -0.024 | 0.347 | 0.025 | 0.846 | 0.025 | 0.133 | 0.032 | 0.628 |
| M5 | -0.667 | 0.000 | 0.300 | 0.663 | -0.222 | 0.587 | 0.456 | 0.042 | 0.133 | 0.027 | 0.667 | 0.000 |
| M6 | -0.637 | 0.124 | -0.047 | 0.631 | -0.033 | 0.851 | 0.368 | 0.485 | 0.348 | 0.900 | 0.637 | 0.124 |
| M7 | -0.125 | 0.005 | -0.184 | 0.773 | -0.326 | 0.339 | -0.076 | 0.306 | 0.710 | 0.020 | 0.125 | 0.005 |
| M8 | 0.519 | 0.972 | 0.315 | 0.963 | -0.218 | 0.490 | -0.201 | 0.909 | -0.415 | 0.623 | -0.519 | 0.972 |
| M9 | 0.106 | 0.431 | 0.702 | 0.831 | -0.298 | 0.717 | -0.206 | 0.464 | -0.304 | 0.840 | -0.106 | 0.431 |
| M10 | 0.369 | 0.440 | 0.194 | 0.516 | -0.457 | 0.960 | -0.286 | 0.774 | 0.181 | 0.908 | -0.369 | 0.440 |
| M11 | -0.319 | 0.918 | 0.195 | 0.938 | -0.064 | 0.633 | 0.259 | 0.921 | -0.071 | 0.445 | 0.319 | 0.918 |
| M12 | -0.643 | 0.492 | 0.342 | 0.181 | -0.248 | 0.488 | 0.157 | 0.775 | 0.391 | 0.828 | 0.643 | 0.492 |
| M13 | -0.028 | 0.334 | -0.004 | 0.896 | -0.016 | 0.312 | 0.034 | 0.979 | 0.014 | 0.028 | 0.028 | 0.334 |
| M14 | -0.233 | 0.263 | 0.102 | 0.993 | -0.304 | 0.655 | 0.063 | 0.600 | 0.371 | 0.888 | 0.233 | 0.263 |
| M15 | 0.131 | 0.000 | 0.046 | 0.730 | -0.357 | 0.914 | -0.040 | 0.134 | 0.220 | 0.065 | -0.131 | 0.000 |
| M16 | -0.461 | 0.036 | -0.193 | 0.494 | -0.216 | 0.570 | 0.161 | 0.193 | 0.709 | 0.556 | 0.461 | 0.036 |
| M17 | 0.458 | 0.679 | 0.038 | 0.879 | 0.006 | 0.934 | -0.349 | 0.987 | -0.153 | 0.504 | -0.458 | 0.679 |
| M18 | -0.053 | 0.049 | -0.130 | 0.603 | -0.306 | 0.179 | 0.041 | 0.695 | 0.448 | 0.493 | 0.053 | 0.049 |
| M19 | -0.420 | 0.625 | 0.486 | 0.581 | 0.365 | 0.927 | 0.104 | 0.915 | -0.535 | 0.891 | 0.420 | 0.625 |
| M20 | -0.496 | 0.015 | 0.321 | 0.180 | 0.154 | 0.538 | 0.085 | 0.142 | -0.064 | 0.899 | 0.496 | 0.015 |
| M21 | 0.491 | 0.106 | -0.255 | 0.888 | -0.089 | 0.646 | -0.226 | 0.131 | 0.079 | 0.680 | -0.491 | 0.106 |
| M22 | -0.172 | 0.917 | -0.314 | 0.496 | -0.261 | 0.432 | 0.095 | 0.880 | 0.651 | 0.074 | 0.172 | 0.917 |
| M23 | -0.226 | 0.012 | 0.375 | 0.485 | -0.398 | 0.670 | 0.171 | 0.089 | 0.077 | 0.059 | 0.226 | 0.012 |
| M24 | -0.096 | 0.115 | 0.042 | 0.070 | -0.270 | 0.338 | -0.019 | 0.535 | 0.343 | 0.077 | 0.096 | 0.115 |
| M25 | 0.103 | 0.441 | 0.434 | 0.763 | -0.075 | 0.988 | -0.273 | 0.822 | -0.190 | 0.823 | -0.103 | 0.441 |
| M26 | 0.042 | 0.499 | -0.275 | 0.160 | -0.048 | 0.980 | -0.218 | 0.925 | 0.499 | 0.527 | -0.042 | 0.499 |
| M27 | -0.436 | 0.002 | 0.155 | 0.959 | -0.328 | 0.411 | -0.009 | 0.078 | 0.618 | 0.051 | 0.436 | 0.002 |
| M28 | 0.373 | 0.110 | 0.229 | 0.967 | -0.143 | 0.796 | -0.152 | 0.195 | -0.307 | 0.561 | -0.373 | 0.110 |
| M29 | -0.471 | 0.026 | -0.029 | 0.459 | -0.372 | 0.631 | 0.215 | 0.459 | 0.657 | 0.097 | 0.471 | 0.026 |

Table S3. 397 significant differential expression proteins and related clusters

| **Cluster 1** | | | | | **Cluster 2** | | | | **Cluster 3** | | | | **Cluster 4** | |
| --- | --- | --- | --- | --- | --- | --- | --- | --- | --- | --- | --- | --- | --- | --- |
| Accession | Gene | Accession | | Gene | Accession | Gene | Accession | Gene | Accession | Gene | Accession | Gene | Accession | Gene |
| A0A075B6K4 | IGLV3-10 | | Q14393 | GAS6 | A0A075B6H7 | IGKV3-7 | P27169 | PON1 | A0A087WTJ2 | GIMAP1-GIMAP5 | Q9BW83 | IFT27 | A0A087WUB9 | CTNNBL1 |
| A0A096LPE2 | SAA2-SAA4 | | Q15113 | PCOLCE | A0A0A0MRZ8 | IGKV3D-11 | P28065 | PSMB9 | A0A087WXI5 | CDH1 | Q9BZZ2 | SIGLEC1 | A0A0G2JN34 | TRPM1 |
| A0A0A0MQS9 | LAMA4 | | Q16822 | PCK2 | A0A0A0MS15 | IGHV3-49 | P28676 | GCA | A0A087WXW9 | COL5A1 | Q9H5N1 | RABEP2 | A0A1W2PNX8 | UNC45A |
| A0A0A0MT60 | FKBP15 | | Q6PCB0 | VWA1 | A0A0A0MTJ9 | NCEH1 | P29466 | CASP1 | A0A0B4J1R7 | BORCS7-ASMT | Q9HBH1 | PDF | A6NC98-3 | CCDC88B |
| A0A0B4J231 | IGLL5 | | Q6PJI9 | WDR59 | A0A0B4J1V0 | IGHV3-15 | P30040 | ERP29 | A0A0C4DFN3 | MGLL | Q9NRX4 | PHPT1 | A6NIX2 | WTIP |
| A0A0C4DFX3 | EMILIN1 | | Q6UVY6 | MOXD1 | A0A0B4J2B5 | IGHV3OR16-9 | P31146 | CORO1A | A0A140T9T7 | TAP1 | Q9NSK0-3 | KLC4 | B5ME19 | EIF3CL |
| A0A0C4DH31 | IGHV1-18 | | Q6ZMJ4 | IL34 | A0A0C4DH25 | IGKV3D-20 | P33241-3 | LSP1 | A0A182DWE9 | HLA-DPB1 | Q9NZL9 | MAT2B | E7ESA6 | PTK2 |
| A0A0C4DH47 | TBXAS1 | | Q6ZMZ3 | SYNE3 | A0A0G2JHC2 | PPP1R18 | P35625 | TIMP3 | A2RUF3 | SRGAP2 | Q9UBS4 | DNAJB11 | E7EVA0 | MAP4 |
| A0A0D5XQ77 | HLA-DQB1 | | Q6ZS17-4 | RIPOR1 | A0A0G2JMH6 | HLA-DRA | P35858-2 | IGFALS | B1B0D4 | ADAMTSL2 | Q9UBX1 | CTSF | E7EX88 | ACAN |
| B0YIW2 | APOC3 | | Q8NES3 | LFNG | A1L4H1 | SSC5D | P36955 | SERPINF1 | B4DDJ5 | MSR1 | Q9UEY8 | ADD3 | F5H5D3 | TUBA1C |
| C9J6P4 | ZC3HAV1 | | Q96T51 | RUFY1 | B1ALD9 | POSTN | P39019 | RPS19 | C9JV77 | AHSG | Q9UKZ9 | PCOLCE2 | G5E9D4 | ELP4 |
| C9JIF9 | APEH | | Q96TA1 | FAM129B | B4DPQ0 | C1R | P39900 | MMP12 | M0R2L9 | RPS19 | Q9Y240 | CLEC11A | K7ES70 | MFAP4 |
| E9PGN7 | SERPING1 | | Q9BVK6 | TMED9 | E9PB61 | ALYREF | P40121 | CAPG | O00300 | TNFRSF11B |  |  | O00487 | PSMD14 |
| G3XAI2 | LAMB1 | | Q9BXR6 | CFHR5 | G3V2W1 | SERPINA10 | P48061-4 | CXCL12 | O00622 | CYR61 |  |  | O14907 | TAX1BP3 |
| G5EA09 | SDCBP | | Q9HD20 | ATP13A1 | H0YAC1 | KLKB1 | P50151 | GNG10 | O43854 | EDIL3 |  |  | O14974 | PPP1R12A |
| H0Y300 | HP | | Q9HDC9 | APMAP | H3BN02 | ITGAX | P51659 | HSD17B4 | O75462 | CRLF1 |  |  | O15355 | PPM1G |
| H0Y8X4 | DNPH1 | | Q9NR99 | MXRA5 | J3KNB4 | CAMP | P52209 | PGD | O75900-2 | MMP23A |  |  | O43294 | TGFB1I1 |
| I3L504 | EIF5A | | Q9NRN5 | OLFML3 | O00299 | CLIC1 | P55058 | PLTP | O94769 | ECM2 |  |  | O75820 | ZNF189 |
| O15344 | MID1 | | Q9P2B2 | PTGFRN | O00391 | QSOX1 | P59998-3 | ARPC4 | O95084 | PRSS23 |  |  | O94875-10 | SORBS2 |
| O43866 | CD5L | | Q9UEU0 | VTI1B | O00533-2 | CHL1 | P61106 | RAB14 | O95236 | APOL3 |  |  | O94875-7 | SORBS2 |
| O95202 | LETM1 | | Q9Y315 | DERA | O14773 | TPP1 | P61626 | LYZ | O95336 | PGLS |  |  | P07305 | H1F0 |
| O95466-3 | FMNL1 | | Q9Y3Z3 | SAMHD1 | O14791-2 | APOL1 | P78324-2 | SIRPA | P00750 | PLAT |  |  | P07951 | TPM2 |
| P00734 | F2 | | Q9Y5K8 | ATP6V1D | O15127 | SCAMP2 | P78417 | GSTO1 | P02462 | COL4A1 |  |  | P09493-8 | TPM1 |
| P00748 | F12 | | V9GYM3 | APOA2 | O43508-2 | TNFSF12 | P80108 | GPLD1 | P02746 | C1QB |  |  | P10915 | HAPLN1 |
| P01024 | C3 | |  |  | O60568 | PLOD3 | Q02809-2 | PLOD1 | P02747 | C1QC |  |  | P12081 | HARS |
| P01031 | C5 | |  |  | O60749 | SNX2 | Q06033 | ITIH3 | P02751-15 | FN1 |  |  | P13611 | VCAN |
| P01042-2 | KNG1 | |  |  | O75348 | ATP6V1G1 | Q08380 | LGALS3BP | P03973 | SLPI |  |  | P13611-2 | VCAN |
| P01602 | IGKV1-5 | |  |  | O75427 | LRCH4 | Q13103 | SPP2 | P04264 | KRT1 |  |  | P19338 | NCL |
| P01619 | IGKV3-20 | |  |  | O95445 | APOM | Q13217 | DNAJC3 | P04440 | HLA-DPB1 |  |  | P21695 | GPD1 |
| P01834 | IGKC | |  |  | P00390 | GSR | Q13576 | IQGAP2 | P05114 | HMGN1 |  |  | P24844 | MYL9 |
| P01860 | IGHG3 | |  |  | P00747 | PLG | Q14019 | COTL1 | P05362 | ICAM1 |  |  | P26447 | S100A4 |
| P02452 | COL1A1 | |  |  | P01019 | AGT | Q15651 | HMGN3 | P05783 | KRT18 |  |  | P29536 | LMOD1 |
| P02549 | SPTA1 | |  |  | P01023 | A2M | Q15833-3 | STXBP2 | P05787-2 | KRT8 |  |  | P35580-3 | MYH10 |
| P02743 | APCS | |  |  | P01591 | JCHAIN | Q641Q3 | METRNL | P07204 | THBD |  |  | P35749 | MYH11 |
| P02748 | C9 | |  |  | P01876 | IGHA1 | Q68D91 | MBLAC2 | P08493-2 | MGP |  |  | P37802-2 | TAGLN2 |
| P02768 | ALB | |  |  | P02647 | APOA1 | Q6STE5 | SMARCD3 | P08572 | COL4A2 |  |  | P46821 | MAP1B |
| P02787 | TF | |  |  | P02649 | APOE | Q6UWH4-2 | FAM198B | P09237 | MMP7 |  |  | P46937-9 | YAP1 |
| P02792 | FTL | |  |  | P02671 | FGA | Q6UY14-3 | ADAMTSL4 | P13645 | KRT10 |  |  | P49747 | COMP |
| P04217 | A1BG | |  |  | P02675 | FGB | Q70UQ0 | IKBIP | P13747 | HLA-E |  |  | P50238 | CRIP1 |
| P04275 | VWF | |  |  | P02753 | RBP4 | Q7Z4W1 | DCXR | P14207 | FOLR2 |  |  | P50461 | CSRP3 |
| P04839 | CYBB | |  |  | P02766 | TTR | Q86UD1 | OAF | P16035 | TIMP2 |  |  | P50914 | RPL14 |
| P05164-3 | MPO | |  |  | P02774-3 | GC | Q8IV08 | PLD3 | P19827 | ITIH1 |  |  | P51911 | CNN1 |
| P05452 | CLEC3B | |  |  | P04003 | C4BPA | Q8IVL6 | P3H3 | P20701-2 | ITGAL |  |  | P62736 | ACTA2 |
| P05546 | SERPIND1 | |  |  | P04004 | VTN | Q8IWU6 | SULF1 | P21589 | NT5E |  |  | P62906 | RPL10A |
| P05997 | COL5A2 | |  |  | P04070-2 | PROC | Q8IY17-4 | PNPLA6 | P21810 | BGN |  |  | P68133 | ACTA1 |
| P06312 | IGKV4-1 | |  |  | P04114 | APOB | Q8N2K0-2 | ABHD12 | P27918 | CFP |  |  | Q00765 | REEP5 |
| P06753-5 | TPM3 | |  |  | P05107 | ITGB2 | Q8NF37 | LPCAT1 | P29279 | CTGF |  |  | Q05682 | CALD1 |
| P07225 | PROS1 | |  |  | P06727 | APOA4 | Q96CG8 | CTHRC1 | P30484 | HLA-B |  |  | Q06787 | FMR1 |
| P07357 | C8A | |  |  | P07093-3 | SERPINE2 | Q96PD5-2 | PGLYRP2 | P35612 | ADD2 |  |  | Q0ZGT2-2 | NEXN |
| P07358 | C8B | |  |  | P07686 | HEXB | Q96RP7 | GAL3ST4 | P41219-2 | PRPH |  |  | Q15124 | PGM5 |
| P07360 | C8G | |  |  | P07741 | APRT | Q9BS26 | ERP44 | P42785-2 | PRCP |  |  | Q15417 | CNN3 |
| P07996 | THBS1 | |  |  | P08603 | CFH | Q9BXX0 | EMILIN2 | P51888 | PRELP |  |  | Q16527 | CSRP2 |
| P08123 | COL1A2 | |  |  | P09467 | FBP1 | Q9H0S4 | DDX47 | P55001 | MFAP2 |  |  | Q562R1 | ACTBL2 |
| P0DOY2 | IGLC2 | |  |  | P09601 | HMOX1 | Q9H2A7 | CXCL16 | P60174 | TPI1 |  |  | Q5VV89 | MGST3 |
| P10451 | SPP1 | |  |  | P09871 | C1S | Q9H8L6 | MMRN2 | Q08431 | MFGE8 |  |  | Q5XXA6 | ANO1 |
| P10586 | PTPRF | |  |  | P10253 | GAA | Q9NQ79 | CRTAC1 | Q10589 | BST2 |  |  | Q6NZI2 | PTRF |
| P10643 | C7 | |  |  | P10319 | HLA-B | Q9NVM1 | EVA1B | Q13332 | PTPRS |  |  | Q6ZR64 | MXRA7 |
| P13671 | C6 | |  |  | P10909-2 | CLU | Q9NX00 | TMEM160 | Q14767 | LTBP2 |  |  | Q7Z7L8 | C11orf96 |
| P14543 | NID1 | |  |  | P11215-2 | ITGAM | Q9NY15 | STAB1 | Q15363 | TMED2 |  |  | Q86WC4 | OSTM1 |
| P18031 | PTPN1 | |  |  | P11498 | PC | Q9UBR2 | CTSZ | Q4G1C9-6 | GLIPR1L2 |  |  | Q8WX93 | PALLD |
| P20700 | LMNB1 | |  |  | P13612 | ITGA4 | Q9UBV2 | SEL1L | Q52LD8 | RFTN2 |  |  | Q92765 | FRZB |
| P28799 | GRN | |  |  | P13796 | LCP1 | Q9UQ80 | PA2G4 | Q86VB7-2 | CD163 |  |  | Q99972 | MYOC |
| P28838 | LAP3 | |  |  | P14618 | PKM | Q9Y646 | CPQ | Q8IY33 | MICALL2 |  |  | Q9BQB4-2 | SOST |
| P35908 | KRT2 | |  |  | P14618-2 | PKM | Q9Y6R7 | FCGBP | Q8ND24 | RNF214 |  |  | Q9BTT0 | ANP32E |
| P36980 | CFHR2 | |  |  | P15144 | ANPEP | R4GN08 | ARPC4 | Q8TEA8 | DTD1 |  |  | Q9BUP0 | EFHD1 |
| P51159 | RAB27A | |  |  | P15153 | RAC2 |  |  | Q92743 | HTRA1 |  |  | Q9BY89 | KIAA1671 |
| P51571 | SSR4 | |  |  | P15169 | CPN1 |  |  | Q92859 | NEO1 |  |  | Q9NP98 | MYOZ1 |
| P55774 | CCL18 | |  |  | P19823 | ITIH2 |  |  | Q96IJ6-2 | GMPPA |  |  | Q9NR12-2 | PDLIM7 |
| P59665 | DEFA1 | |  |  | P19971 | TYMP |  |  | Q96P44 | COL21A1 |  |  | Q9P0L0-2 | VAPA |
| P83111 | LACTB | |  |  | P21281 | ATP6V1B2 |  |  | Q96P47-4 | AGAP3 |  |  | Q9UBP9 | GULP1 |
| P98171-2 | ARHGAP4 | |  |  | P21399 | ACO1 |  |  | Q96S66 | CLCC1 |  |  | Q9UDY4 | DNAJB4 |
| Q03591 | CFHR1 | |  |  | P24821 | TNC |  |  | Q99470 | SDF2 |  |  | Q9UGI8 | TES |
| Q07507 | DPT | |  |  | P26572 | MGAT1 |  |  | Q9BRK3-2 | MXRA8 |  |  | Q9UNF0 | PACSIN2 |
| Q13162 | PRDX4 | |  |  | P27105 | STOM |  |  | Q9BVT8 | TMUB1 |  |  | Q9Y2X7-3 | GIT1 |

Table S4. A list of 51 DEPs associated with complement

| Gene | Accession | Cluster | N | PIT | FA | TCFA | RP |
| --- | --- | --- | --- | --- | --- | --- | --- |
| A2M | P01023 | 2 | 1 | 1.33875 | 1.34225 | 1.71725 | 1.88725 |
| ALB | P02768 | 1 | 1 | 1.29325 | 1.40625 | 1.56925 | 1.78425 |
| APCS | P02743 | 1 | 1 | 1.4215 | 1.488 | 1.494 | 1.7845 |
| APOA1 | P02647 | 2 | 1 | 1.19325 | 1.16925 | 1.639 | 1.7835 |
| C1QB | P02746 | 3 | 1 | 1.53925 | 1.25675 | 1.609 | 1.58225 |
| C1QC | P02747 | 3 | 1 | 1.6255 | 1.239 | 1.65425 | 1.57875 |
| C1R | B4DPQ0 | 2 | 1 | 1.187 | 1.15425 | 1.4175 | 1.315 |
| C1S | P09871 | 2 | 1 | 1.225 | 1.16525 | 1.3985 | 1.3795 |
| C3 | P01024 | 1 | 1 | 1.177 | 1.35725 | 1.417 | 1.64875 |
| C4BPA | P04003 | 2 | 1 | 1.544 | 1.3415 | 1.90475 | 1.94425 |
| C5 | P01031 | 1 | 1 | 1.52925 | 1.6205 | 2.1175 | 2.6195 |
| C6 | P13671 | 1 | 1 | 1.31025 | 1.3905 | 1.67375 | 1.812 |
| C7 | P10643 | 1 | 1 | 1.35 | 1.42025 | 1.762 | 2.10475 |
| C8A | P07357 | 1 | 1 | 1.2695 | 1.21375 | 1.43625 | 1.7195 |
| C8B | P07358 | 1 | 1 | 1.3815 | 1.31025 | 1.5275 | 1.69125 |
| C8G | P07360 | 1 | 1 | 1.37675 | 1.2935 | 1.589 | 2.026 |
| C9 | P02748 | 1 | 1 | 1.63825 | 1.612 | 1.8605 | 2.33325 |
| CFH | P08603 | 2 | 1 | 1.26525 | 1.3035 | 1.472 | 1.5215 |
| CFHR1 | Q03591 | 1 | 1 | 1.48275 | 1.54 | 1.60425 | 1.81775 |
| CFHR2 | P36980 | 1 | 1 | 1.23475 | 1.15325 | 1.3285 | 1.8105 |
| CFHR5 | Q9BXR6 | 1 | 1 | 1.482667 | 1.252667 | 1.601 | 2.846667 |
| CFP | P27918 | 3 | 1 | 1.69625 | 1.3815 | 1.52725 | 1.37075 |
| CLU | P10909-2 | 2 | 1 | 1.435 | 1.58175 | 1.9745 | 2.0285 |
| CPN1 | P15169 | 2 | 1 | 1.391 | 1.253 | 1.63225 | 1.66175 |
| F2 | P00734 | 1 | 1 | 1.2725 | 1.44225 | 1.38325 | 1.4905 |
| FGA | P02671 | 2 | 1 | 1.507 | 1.234 | 1.7475 | 1.91975 |
| FGB | P02675 | 2 | 1 | 1.70725 | 1.3375 | 1.79675 | 1.87625 |
| GAS6 | Q14393 | 1 | 1 | 1.307667 | 1.283667 | 1.305333 | 1.526667 |
| ICAM1 | P05362 | 3 | 1 | 1.88225 | 2.08725 | 2.41375 | 1.806 |
| ITGAM | P11215-2 | 2 | 1 | 1.4185 | 1.378 | 1.78625 | 1.663 |
| ITGAX | H3BN02 | 2 | 1 | 1.387 | 1.41475 | 1.9415 | 1.80075 |
| ITGB2 | P05107 | 2 | 1 | 1.63925 | 1.64325 | 2.1695 | 2.3 |
| KLKB1 | H0YAC1 | 2 | 1 | 1.2645 | 1.17475 | 1.67325 | 1.89325 |
| LAMB1 | G3XAI2 | 1 | 1 | 1.2115 | 1.47575 | 1.503 | 1.62975 |
| MFGE8 | Q08431 | 3 | 1 | 1.36075 | 1.14325 | 1.31925 | 1.21425 |
| PLG | P00747 | 2 | 1 | 1.27925 | 1.2275 | 1.6335 | 1.563 |
| PROS1 | P07225 | 1 | 1 | 1.3875 | 1.14175 | 1.24475 | 1.68775 |
| RPS19 | P39019 | 2 | 1 | 1.01975 | 1.15525 | 1.30625 | 1.24475 |
| RPS19 | M0R2L9 | 3 | 1 | 1.70875 | 1.6825 | 2.109 | 1.33675 |
| SERPING1 | E9PGN7 | 1 | 1 | 1.134667 | 1.347333 | 1.272667 | 1.514333 |
| SPP1 | P10451 | 1 | 1 | 1.6015 | 1.23175 | 1.5385 | 1.94375 |
| THBS1 | P07996 | 1 | 1 | 1.3835 | 1.50675 | 1.82925 | 2.0315 |
| VTN | P04004 | 2 | 1 | 1.52775 | 1.52375 | 2.08975 | 2.12725 |
| F12 | P00748 | 1 | 1 | 1.203 | 1.331667 | 1.312333 | 1.502 |
| VWF | P04275 | 1 | 1 | 1.18725 | 1.28775 | 1.35925 | 1.4255 |
| THBD | P07204 | 3 | 1 | 1.411 | 1.4025 | 1.37925 | 1.32575 |
| PROC | P04070-2 | 2 | 1 | 1.41775 | 1.59325 | 1.87925 | 1.735 |
| SERPINE2 | P07093-3 | 2 | 1 | 1.525333 | 1.499 | 2.551667 | 1.866333 |
| PLAT | P00750 | 3 | 1 | 1.624 | 1.709 | 1.63875 | 1.51975 |
| KNG1 | P01042-2 | 1 | 1 | 1.359 | 1.482 | 1.5865 | 1.6625 |
| SERPIND1 | P05546 | 1 | 1 | 1.139333 | 1.370333 | 1.601333 | 2.193667 |

Table S5. A list of 85 DEPs associated with ECM

| Accession | Gene | Cluster | Module | N | PIT | FA | TCFA | RP |
| --- | --- | --- | --- | --- | --- | --- | --- | --- |
| A0A0A0MQS9 | LAMA4 | 1 | 30 | 1 | 1.06775 | 1.29 | 1.251 | 1.37075 |
| A0A0C4DFX3 | EMILIN1 | 1 | 18 | 1 | 1.1725 | 1.25425 | 1.32125 | 1.39025 |
| E9PGN7 | SERPING1 | 1 | 15 | 1 | 1.134667 | 1.347333 | 1.272667 | 1.514333 |
| G3XAI2 | LAMB1 | 1 | 7 | 1 | 1.2115 | 1.47575 | 1.503 | 1.62975 |
| P00734 | F2 | 1 | 3 | 1 | 1.2725 | 1.44225 | 1.38325 | 1.4905 |
| P00748 | F12 | 1 | 15 | 1 | 1.203 | 1.331667 | 1.312333 | 1.502 |
| P01042-2 | KNG1 | 1 | 15 | 1 | 1.359 | 1.482 | 1.5865 | 1.6625 |
| P02452 | COL1A1 | 1 | 18 | 1 | 1.4355 | 1.76925 | 1.468 | 1.69775 |
| P04275 | VWF | 1 | 5 | 1 | 1.18725 | 1.28775 | 1.35925 | 1.4255 |
| P05452 | CLEC3B | 1 | 18 | 1 | 1.12075 | 1.653 | 1.30375 | 1.49225 |
| P05546 | SERPIND1 | 1 | 23 | 1 | 1.139333 | 1.370333 | 1.601333 | 2.193667 |
| P05997 | COL5A2 | 1 | 3 | 1 | 1.41 | 1.58975 | 1.6305 | 1.71925 |
| P07996 | THBS1 | 1 | 5 | 1 | 1.3835 | 1.50675 | 1.82925 | 2.0315 |
| P08123 | COL1A2 | 1 | 18 | 1 | 1.56325 | 1.8535 | 1.67575 | 2.05275 |
| P10451 | SPP1 | 1 | 7 | 1 | 1.6015 | 1.23175 | 1.5385 | 1.94375 |
| P14543 | NID1 | 1 | 18 | 1 | 1.17525 | 1.2715 | 1.299 | 1.31875 |
| P55774 | CCL18 | 1 | 15 | 1 | 1.154667 | 1.396667 | 1.341333 | 1.642333 |
| Q07507 | DPT | 1 | 18 | 1 | 1.23875 | 1.303 | 1.27725 | 1.35875 |
| Q14393 | GAS6 | 1 | 29 | 1 | 1.307667 | 1.283667 | 1.305333 | 1.526667 |
| Q15113 | PCOLCE | 1 | 29 | 1 | 1.0185 | 1.266 | 1.31275 | 1.31175 |
| Q6PCB0 | VWA1 | 1 | 27 | 1 | 1.2605 | 1.25275 | 1.33475 | 1.41575 |
| Q6ZMJ4 | IL34 | 1 | 1 | 1 | 1.302667 | 1.297 | 1.215333 | 1.395333 |
| Q9NR99 | MXRA5 | 1 | 27 | 1 | 1.03975 | 1.079 | 1.58175 | 1.83875 |
| B1ALD9 | POSTN | 2 | 29 | 1 | 1.25775 | 1.39875 | 1.5095 | 1.463 |
| G3V2W1 | SERPINA10 | 2 | 27 | 1 | 1.122 | 0.947667 | 1.756 | 1.817333 |
| O43508-2 | TNFSF12 | 2 | 15 | 1 | 1.6065 | 1.93625 | 2.45375 | 2.341 |
| O60568 | PLOD3 | 2 | 3 | 1 | 1.262 | 1.127 | 1.32375 | 1.344 |
| P00747 | PLG | 2 | 23 | 1 | 1.27925 | 1.2275 | 1.6335 | 1.563 |
| P01019 | AGT | 2 | 5 | 1 | 1.278 | 1.265 | 1.472333 | 1.506667 |
| P01023 | A2M | 2 | 5 | 1 | 1.33875 | 1.34225 | 1.71725 | 1.88725 |
| P02671 | FGA | 2 | 5 | 1 | 1.507 | 1.234 | 1.7475 | 1.91975 |
| P02675 | FGB | 2 | 5 | 1 | 1.70725 | 1.3375 | 1.79675 | 1.87625 |
| P04004 | VTN | 2 | 5 | 1 | 1.52775 | 1.52375 | 2.08975 | 2.12725 |
| P07093-3 | SERPINE2 | 2 | 3 | 1 | 1.525333 | 1.499 | 2.551667 | 1.866333 |
| P19823 | ITIH2 | 2 | 23 | 1 | 1.46175 | 1.40525 | 1.99475 | 1.9165 |
| P24821 | TNC | 2 | 15 | 1 | 1.355333 | 1.423 | 1.760667 | 1.657667 |
| P35625 | TIMP3 | 2 | 5 | 1 | 1.08575 | 1.11075 | 1.3375 | 1.3545 |
| P35858-2 | IGFALS | 2 | 5 | 1 | 1.33125 | 1.23975 | 1.61825 | 1.667 |
| P36955 | SERPINF1 | 2 | 23 | 1 | 1.1515 | 1.28775 | 1.547 | 1.595 |
| P39900 | MMP12 | 2 | 3 | 1 | 1.6485 | 1.51525 | 2.1705 | 2.15575 |
| P48061-4 | CXCL12 | 2 | 15 | 1 | 1.39175 | 1.54225 | 1.804 | 1.82875 |
| Q02809-2 | PLOD1 | 2 | 5 | 1 | 1.120667 | 1.157333 | 1.673333 | 1.424333 |
| Q06033 | ITIH3 | 2 | 27 | 1 | 1.495 | 1.165667 | 2.032333 | 1.719667 |
| Q6UY14-3 | ADAMTSL4 | 2 | 3 | 1 | 1.2455 | 1.224 | 1.3935 | 1.32 |
| Q8IWU6 | SULF1 | 2 | 5 | 1 | 1.34025 | 1.268 | 1.5355 | 1.50325 |
| Q96CG8 | CTHRC1 | 2 | 14 | 1 | 1.418 | 1.4425 | 1.62475 | 1.612 |
| Q9BXX0 | EMILIN2 | 2 | 15 | 1 | 1.39875 | 1.4975 | 1.86725 | 2.005 |
| Q9H8L6 | MMRN2 | 2 | 27 | 1 | 1.25225 | 1.371 | 1.4975 | 1.509 |
| Q9UBR2 | CTSZ | 2 | 23 | 1 | 1.41 | 1.4025 | 1.88675 | 1.7835 |
| A0A087WXW9 | COL5A1 | 3 | 18 | 1 | 2.06225 | 2.39275 | 1.97725 | 2.40675 |
| B1B0D4 | ADAMTSL2 | 3 | 34 | 1 | 1.541 | 1.12975 | 1.397 | 1.4965 |
| O00622 | CYR61 | 3 | 10 | 1 | 1.532 | 1.3525 | 1.315 | 1.4265 |
| O43854 | EDIL3 | 3 | 34 | 1 | 1.407 | 1.20975 | 1.45175 | 1.4275 |
| O75462 | CRLF1 | 3 | 20 | 1 | 1.561 | 1.31975 | 1.3885 | 1.42825 |
| O94769 | ECM2 | 3 | 23 | 1 | 1.43025 | 1.59475 | 1.64625 | 1.5515 |
| P00750 | PLAT | 3 | 15 | 1 | 1.624 | 1.709 | 1.63875 | 1.51975 |
| P02462 | COL4A1 | 3 | 18 | 1 | 1.33625 | 1.39575 | 1.39825 | 1.39475 |
| P02746 | C1QB | 3 | 3 | 1 | 1.53925 | 1.25675 | 1.609 | 1.58225 |
| P02747 | C1QC | 3 | 3 | 1 | 1.6255 | 1.239 | 1.65425 | 1.57875 |
| P02751-15 | FN1 | 3 | 21 | 1 | 1.2835 | 1.2175 | 1.30275 | 1.208 |
| P03973 | SLPI | 3 | 0 | 1 | 1.26825 | 1.4665 | 1.7655 | 1.1985 |
| P08493-2 | MGP | 3 | 16 | 1 | 1.9915 | 1.79725 | 1.5615 | 1.40575 |
| P08572 | COL4A2 | 3 | 18 | 1 | 1.3655 | 1.61875 | 1.388 | 1.32025 |
| P09237 | MMP7 | 3 | 0 | 1 | 2.525667 | 1.158667 | 1.884 | 2.080667 |
| P16035 | TIMP2 | 3 | 0 | 1 | 1.099 | 1.152 | 1.412 | 1.06075 |
| P19827 | ITIH1 | 3 | 7 | 1 | 1.499 | 1.35675 | 1.588 | 1.54275 |
| P21810 | BGN | 3 | 15 | 1 | 1.312 | 1.286 | 1.37275 | 1.275 |
| P29279 | CTGF | 3 | 15 | 1 | 1.453667 | 1.418333 | 1.477333 | 1.384 |
| P51888 | PRELP | 3 | 5 | 1 | 1.21875 | 1.27325 | 1.318 | 1.27525 |
| P55001 | MFAP2 | 3 | 21 | 1 | 1.22575 | 1.181 | 1.31025 | 1.21625 |
| Q08431 | MFGE8 | 3 | 20 | 1 | 1.36075 | 1.14325 | 1.31925 | 1.21425 |
| Q14767 | LTBP2 | 3 | 27 | 1 | 1.3375 | 1.3605 | 1.37375 | 1.20475 |
| Q92743 | HTRA1 | 3 | 5 | 1 | 1.27725 | 1.15725 | 1.34575 | 1.24425 |
| Q96P44 | COL21A1 | 3 | 7 | 1 | 1.38825 | 1.33375 | 1.37675 | 1.2985 |
| Q9UBX1 | CTSF | 3 | 23 | 1 | 1.495 | 1.5615 | 1.77 | 1.68575 |
| Q9UKZ9 | PCOLCE2 | 3 | 15 | 1 | 1.432333 | 1.460667 | 1.756 | 1.387 |
| Q9Y240 | CLEC11A | 3 | 3 | 1 | 1.43425 | 1.2995 | 1.6225 | 1.4995 |
| E7EX88 | ACAN | 4 | 24 | 1 | 1.4955 | 0.96775 | 0.75325 | 0.749 |
| K7ES70 | MFAP4 | 4 | 20 | 1 | 0.56425 | 0.53675 | 0.562 | 0.64525 |
| P10915 | HAPLN1 | 4 | 24 | 1 | 1.24725 | 0.8415 | 0.78775 | 0.68925 |
| P13611 | VCAN | 4 | 16 | 1 | 0.58925 | 0.6465 | 0.523 | 0.462 |
| P13611-2 | VCAN | 4 | 3 | 1 | 0.77975 | 0.91525 | 0.62475 | 0.58775 |
| P26447 | S100A4 | 4 | 20 | 1 | 0.76725 | 0.94375 | 0.7875 | 0.7905 |
| P49747 | COMP | 4 | 8 | 1 | 0.655 | 0.634 | 0.641 | 0.763 |
| Q92765 | FRZB | 4 | 1 | 1 | 0.84275 | 0.89625 | 0.7575 | 0.66275 |

Table S6. Clinical characteristics of the discovery plasma proteomics cohort.

|  | CAG negative | CAG positive (n=40) | | P-value |
| --- | --- | --- | --- | --- |
|  | (n=40) | Stable CAD(n=18) | ACS  (n=22) |  |
| **Demographics** |  |  |  |  |
| Age, years | 65.60 ± 9.85 | 67.06 ± 11.21 | 70.18 ± 10.28 | 0.25 |
| Sex (male) | 23 (57.50%) | 15 (83.33%) | 16 (72.73%) | 0.125 |
| **Comorbidity and risk factors** | |  |  |  |
| Hypertension | 21 (52.50%) | 14 (77.78%) | 19 (86.36%) | 0.014 |
| SBP, mmHg | 122.38 ± 19.70 | 132.69 ± 13.99 | 128.14 ± 19.17 | 0.167 |
| DBP, mmHg | 71.32 ± 11.89 | 75.44 ± 11.52 | 72.77 ± 11.77 | 0.518 |
| Diabetes | 7 (17.50%) | 7 (38.89%) | 7 (31.82%) | 0.181 |
| Chronic kidney disease | 1 (2.50%) | 1 (5.56%) | 1 (4.55%) |  |
| Smoking active | 4 (10.00%) | 6 (33.33%) | 8 (36.36%) | 0.027 |
| Drinking | 7 (17.50%) | 3 (16.67%) | 4 (18.18%) | 0.992 |
| Family history of CAD | 1 (2.50%) | 1 (5.56%) | 2 (9.09%) | 0.441 |
| **Laboratory parameters** |  |  |  |  |
| Fasting glucose, mmol/L | 5.77 ± 1.38 | 6.50 ± 3.26 | 6.56 ± 2.67 | 0.402 |
| HbA1C | 5.94 ± 0.71 | 6.17 ± 1.28 | 6.52 ± 2.91 | 0.546 |
| eGFR, mL/min/1.73 m² | 84.27 ± 18.08 | 78.11 ± 23.18 | 76.54 ± 23.96 | 0.347 |
| LDL-C, mmol/L | 2.4 ± 0.75 | 2.81 ± 1.08 | 2.88 ± 0.89 | 0.089 |
| HDL-C, mmol/L | 1.25 ± 0.37 | 1.20 ± 0.28 | 1.16 ± 0.35 | 0.661 |
| TC, mmol/L | 4.13 ± 1.12 | 4.59 ± 1.24 | 4.54 ± 1.11 | 0.24 |
| TG, mmol/L | 1.51 ± 0.83 | 1.79 ± 1.21 | 1.56 ± 1.31 | 0.637 |
| **Severity of CAD** |  |  |  |  |
| 1 vessel | - | 9 (50.00%) | 6 (27.27%) | 0.325 |
| 2 vessels | - | 4 (22.22%) | 8 (36.36%) |  |
| ≥3 vessels | - | 5 (27.78%) | 8 (36.36%) |  |
| SYNTAX score | - | 7.50 (0.00-19.25) | 16.50 (10.25-23.50) | 0.101 |

Continuous data are shown as mean ± SD or median (interquartile range). Dichotomous data are shown as n (%). One way ANOVA was used for the statistical comparison of continuous variables between CAD negative, stable CAD and ACS groups, The Chi-square test or Fisher’s exact test for the categorical variables. SBP, systolic blood pressure; DBP, diastolic blood pressure; TC, total cholesterol; TG, triglycerides; HDL-C, high-density lipoprotein- cholesterol; LDL-C, low-density lipoprotein-cholesterol; Hemoglobin A1C, HbA1C; eGFR, estimated glomerular filtration rate; CAD, coronary artery disease;

Table S7. A list of 65 proteins participated in ECM organization, complement and coagulation cascades pathways

| Proteins |  |
| --- | --- |
| ECM2 | CFH |
| F2 | SPP1 |
| C1R | C7 |
| PLG | CLU |
| F12 | ITGAM |
| PLAT | C6 |
| AGT | CPN1 |
| A2M | ITIH2 |
| C3 | ITIH1 |
| C5 | CFP |
| KNG1 | TIMP3 |
| APOA1 | IGFALS |
| FGA | SERPINF1 |
| FGB | CFHR2 |
| APCS | RPS19 |
| C1QB | CXCL12 |
| C1QC | PRELP |
| C9 | CCL18 |
| ALB | CFHR1 |
| KLKB1 | ITIH3 |
| C4BPA | MFGE8 |
| VTN | LTBP2 |
| PROC | POSTN |
| VWF | PCOLCE |
| ITGB2 | ADAMTSL4 |
| SERPING1 | HTRA1 |
| SERPIND1 | CFHR5 |
| COL5A2 | MMRN2 |
| SERPINE2 | MXRA5 |
| PROS1 | CTSF |
| C8B | SERPINA10 |
| C8G | PCOLCE2 |
| THBS1 |  |

Table S8. Clinical characteristics of the external validation cohort.

|  | CAG negative | CAG positive n=88 | | P-value |
| --- | --- | --- | --- | --- |
|  | n=25 | Stable CAD, n=71 | ACS, n=17 |  |
| Age, years | 67.24±9.57 | 70.99±8.44 | 69.29±8.64 | 0.178 |
| Sex(male) | 10 (40.00%) | 51 (71.83%) | 16 (94.12%) | <0.001 |
| BMI, kg/m^2^ | 24.24 ±3.24 | 24.23±3.81 | 24.81±3.86 | 0.843 |
| rural area | 8 (32.00%) | 19 (26.76%) | 3 (17.65%) | 0.585 |
| **Comorbidity and risk factors** | |  |  |  |
| Hypertension | 8 (32.00%) | 42 (59.15%) | 12 (70.59%) | 0.023 |
| SBP, mmHg | 126.56 ±16.63 | 136.37±20.23 | 140.94±16.41 | 0.034 |
| DBP, mmHg | 73.00±9.21 | 75.96±10.86 | 82.82±13.44 | 0.018 |
| Smoking active | 5 (20.00%) | 23 (32.39%) | 9 (52.94%) | 0.082 |
| Drinking | 3 (12.00%) | 19 (26.76%) | 2 (11.76%) | 0.175 |
| Chronic kidney disease | 0 (0.00%) | 3 (4.23%) | 0 (0.00%) | 0.735 |
| Diabetes | 3 (12.00%) | 21 (29.58%) | 4 (23.53%) | 0.214 |
| Family history of CAD | 4 (16.00%) | 31 (43.66%) | 3 (17.65%) | 0.013 |
| **Laboratory parameters** |  |  |  |  |
| Fasting glucose, mmol/L | 5.16 (4.93-5.63) | 5.60 (5.16-6.50) | 5.50 (5.19-6.59) | 0.159 |
| HBA1C, % | 5.55±1.27 | 6.35 ±1.70 | 6.24 ±1.84 | 0.121 |
| TG, mmol/L | 1.10 (0.88-1.44) | 1.38 (1.04-1.95) | 1.22 (1.02-1.47) | 0.068 |
| TC, mmol/L | 4.01 ±1.38 | 3.99±0.98 | 3.97±0.75 | 0.992 |
| HDL-C, mmol/L | 1.21±0.37 | 1.17 ±0.31 | 1.03 ±0.16 | 0.151 |
| LDL-C, mmol/L | 2.35±1.00 | 2.35±0.82 | 2.52±0.66 | 0.758 |
| Creatinine,μmol/L | 69.00 (66.00-85.00) | 82.00 (69.00-93.00) | 80.00 (70.00-85.00) | 0.165 |
| eGFR, mL/min/1.73 m² | 80.55±20.84 | 82.45±15.10 | 85.14±13.42 | 0.671 |
| **Severity of CAD** |  |  |  | <0.001 |
| none | 25 (100.00%) | 0 (0.00%) | 0 (0.00%) |  |
| 1 vessel | - | 25 (35.21%) | 3 (17.65%) |  |
| 2 vessels | - | 22 (30.99%) | 4 (23.53%) |  |
| ≥3 vessels | - | 24 (33.80%) | 10 (58.82%) |  |
| SYNTAX score | - | 7.00 (3.50-13.75) | 15.25 (12.00-20.00) | <0.001 |

Continuous data are shown as mean ± SD or median (interquartile range). Dichotomous data are shown as n (%). One way ANOVA was used for the statistical comparison of continuous variables between CAD negative, stable CAD and ACS groups, The Chi-square test or Fisher’s exact test for the categorical variables. SBP, systolic blood pressure; DBP, diastolic blood pressure; TC, total cholesterol; TG, triglycerides; HDL-C, high-density lipoprotein- cholesterol; LDL-C, low-density lipoprotein-cholesterol; Hemoglobin A1C, HbA1C; eGFR, estimated glomerular filtration rate; CAD, coronary artery disease;

Table S9. Association between each biomarker and CAD in different subgroups

| Variable | Count | Percent | OR | Lower | Upper | P value | P for interaction |
| --- | --- | --- | --- | --- | --- | --- | --- |
| **THBS1** | | | | | | | |
| **Overall** | 113 | 100 | 1.0003 | 1.0001 | 1.0005 | 0.005 |  |
| **Age** |  |  |  |  |  |  | 0.554 |
| >70 years | 55 | 48.673 | 1.0004 | 1 | 1.001 | 0.045 |  |
| ≤70 years | 58 | 51.327 | 1.0002 | 1 | 1.0005 | 0.03 |  |
| **Sex** |  |  |  |  |  |  | 0.048 |
| Female | 36 | 31.858 | 1.00008 | 0.9998 | 1.0003 | 0.484 |  |
| Male | 77 | 68.142 | 1.0006 | 1.0002 | 1.001 | 0.009 |  |
| **Diabetes** |  |  |  |  |  |  | 0.469 |
| No | 85 | 75.221 | 1.0002 | 1 | 1.0005 | 0.012 |  |
| Yes | 28 | 24.779 | 1.0005 | 0.9999 | 1.0015 | 0.161 |  |
| **eGFR** |  |  |  |  |  |  | 0.138 |
| ≥90mL/min/1.73 m² | 41 | 36.283 | 1.001 | 1.0002 | 1.0012 | 0.021 |  |
| <90mL/min/1.73 m² | 72 | 63.717 | 1.0002 | 1 | 1.0004 | 0.067 |  |
| **Smoke** |  |  |  |  |  |  | 0.521 |
| No | 76 | 67.257 | 1.0004 | 1 | 1.001 | 0.028 |  |
| Yes | 37 | 32.743 | 1.0002 | 1 | 1.0005 | 0.094 |  |
| **Hypertension** |  |  |  |  |  |  | 0.948 |
| No | 51 | 45.133 | 1.0003 | 1.0001 | 1.0005 | 0.029 |  |
| Yes | 62 | 54.867 | 1.0003 | 1 | 1.0007 | 0.087 |  |
| **Diabetes** |  |  |  |  |  |  | 0.469 |
| No | 85 | 75.221 | 1.0002 | 1 | 1.0005 | 0.012 |  |
| Yes | 62 | 54.867 | 1.0003 | 1 | 1.0007 | 0.087 |  |
| **ECM2** | | | | | | | |
| **Overall** | 113 | 100 | 1.004 | 1.002 | 1.007 | 0.002 |  |
| **Age** |  |  |  |  |  |  | 0.186 |
| >70 years | 55 | 48.673 | 1.008 | 1.002 | 1.014 | 0.013 |  |
| ≤70 years | 58 | 51.327 | 1.003 | 1 | 1.006 | 0.048 |  |
| **Sex** |  |  |  |  |  |  | 0.714 |
| Female | 36 | 31.858 | 1.004 | 1 | 1.008 | 0.074 |  |
| Male | 77 | 68.142 | 1.005 | 1.001 | 1.009 | 0.011 |  |
| **Diabetes** |  |  |  |  |  |  | 0.484 |
| No | 85 | 75.221 | 1.005 | 1.001 | 1.008 | 0.006 |  |
| Yes | 28 | 24.779 | 1.007 | 1 | 1.015 | 0.049 |  |
| **eGFR** |  |  |  |  |  |  | 0.032 |
| ≥90mL/min/1.73 m² | 41 | 36.283 | 1.001 | 0.997 | 1.005 | 0.642 |  |
| <90mL/min/1.73 m² | 72 | 63.717 | 1.007 | 1.003 | 1.011 | 0.001 |  |
| **Smoke** |  |  |  |  |  |  | 0.155 |
| No | 76 | 67.257 | 1.003 | 1 | 1.007 | 0.044 |  |
| Yes | 37 | 32.743 | 1.009 | 1.002 | 1.015 | 0.01 |  |
| **Hypertension** |  |  |  |  |  |  | 0.767 |
| No | 51 | 45.133 | 1.006 | 1.001 | 1.01 | 0.009 |  |
| Yes | 62 | 54.867 | 1.005 | 1.001 | 1.009 | 0.021 |  |
| **C1R** | | | | | | | |
| **Overall** | 113 | 100 | 1.216 | 1.071 | 1.381 | 0.002 |  |
| **Age** |  |  |  |  |  |  | 0.187 |
| >70 years | 55 | 48.673 | 1.381 | 1.071 | 1.782 | 0.013 |  |
| ≤70 years | 58 | 51.327 | 1.131 | 0.97 | 1.318 | 0.116 |  |
| **Sex** |  |  |  |  |  |  | 0.213 |
| Female | 36 | 31.858 | 1.127 | 0.952 | 1.333 | 0.165 |  |
| Male | 77 | 68.142 | 1.358 | 1.067 | 1.729 | 0.013 |  |
| **Diabetes** |  |  |  |  |  |  | 0.692 |
| No | 85 | 75.221 | 1.203 | 1.051 | 1.378 | 0.007 |  |
| Yes | 28 | 24.779 | 1.309 | 0.882 | 1.941 | 0.181 |  |
| **eGFR** |  |  |  |  |  |  | 0.173 |
| ≥90mL/min/1.73 m² | 41 | 36.283 | 1.09 | 0.901 | 1.319 | 0.373 |  |
| <90mL/min/1.73 m² | 72 | 63.717 | 1.307 | 1.094 | 1.562 | 0.003 |  |
| **Smoke** |  |  |  |  |  |  | 0.193 |
| No | 76 | 67.257 | 1.17 | 1.022 | 1.341 | 0.023 |  |
| Yes | 37 | 32.743 | 1.84 | 0.944 | 3.587 | 0.073 |  |
| **Hypertension** |  |  |  |  |  |  | 0.351 |
| No | 51 | 45.133 | 1.16 | 0.986 | 1.365 | 0.074 |  |
| Yes | 62 | 54.867 | 1.339 | 1.038 | 1.728 | 0.025 |  |

Abbreviations: OR = Odds Ratio，eGFR = estimated Glomerular Filtration Rate Analysis was conducted in the external validation cohort.

Table S10 Multivariate logistic analysis of the three new biomarkers and presence of CAD in the validation cohort.

| Characteristic | OR | 95% CI | p-value |
| --- | --- | --- | --- |
| THBS1 | 1.001 | 1.000-1.001 | 0.002 |
| C1R | 1.218 | 1.010-1.544 | 0.064 |
| ECM2 | 1.008 | 1.003-1.015 | 0.006 |
| Sex(male) vs female | 6.106 | 1.006-47.920 | 0.061 |
| Age, years | 1.089 | 0.994-1.207 | 0.081 |
| SBP, mmHg | 1.018 | 0.974-1.068 | 0.446 |
| BMI, kg/m^2^ | 0.936 | 0.737-1.179 | 0.570 |
| Diabetes |  |  | 0.070 |
| No | ref |  |  |
| Yes | 8.362 | 1.132-128.865 |  |
| Smoking |  |  | 0.837 |
| No | ref |  |  |
| Yes | 0.794 | 0.081-7.586 |  |
| LDL-C, mmol/L | 2.79 | 0.532-20.208 | 0.224 |
| Total cholesterol, mmol/L | 0.438 | 0.096-1.603 | 0.207 |
| Triglyceride, mmol/L | 9.014 | 1.731-67.177 | 0.018 |
| Creatinine, μmol/L | 0.992 | 0.954-1.032 | 0.690 |

Abbreviations: CI = Confidence Interval, OR = Odds Ratio

Table S11 Comparison of diagnostic performance for CAD between the clinical model and the clinical + novel biomarker model

|  | AUC  95%CI | P value | NRI, 95%CI  (Categorical) | P value | NRI,95%CI  (Continuous) | P value | IDI, 95%CI | P value | |
| --- | --- | --- | --- | --- | --- | --- | --- | --- | --- |
| Model clinical | 0.820  (0.714-0.925) |  |  |  |  |  |  | |  |
| Model  clinical+  biomarker panel | 0.947  (0.906-0.988) | 0.005 | 0.354  (0.128 - 0.580) | 0.002 | 1.191  (0.835-1.547) | <0.001 | 0.227  (0.121- 0.332) | | <0.001 |

Abbreviations: AUC = Area Under the Curve, NRI = Net Reclassification Improvement,

IDI = Integrated Discrimination Improvement

Variables in clinical model include age, sex, systolic blood pressure, diabetes, renal function, smoking, LDL-C, TG, Cystatin C and lipid lowering therapy.

**SUPPLEMENTAL REFERENCES**

1. Stary HC. Natural history and histological classification of atherosclerotic lesions: an update. *Arterioscler Thromb Vasc Biol*. 2000;20:1177-1178. doi: 10.1161/01.atv.20.5.1177

2. Dayon L, Hainard A, Licker V, Turck N, Kuhn K, Hochstrasser DF, Burkhard PR, Sanchez JC. Relative quantification of proteins in human cerebrospinal fluids by MS/MS using 6-plex isobaric tags. *Anal Chem*. 2008;80:2921-2931. doi: 10.1021/ac702422x

3. Chen L, Yang F, Chen X, Rao M, Zhang NN, Chen K, Deng H, Song JP, Hu SS. Comprehensive Myocardial Proteogenomics Profiling Reveals C/EBPalpha as the Key Factor in the Lipid Storage of ARVC. *J Proteome Res*. 2017;16:2863-2876. doi: 10.1021/acs.jproteome.7b00165

4. Zhou Y, Zhou B, Pache L, Chang M, Khodabakhshi AH, Tanaseichuk O, Benner C, Chanda SK. Metascape provides a biologist-oriented resource for the analysis of systems-level datasets. *Nat Commun*. 2019;10:1523. doi: 10.1038/s41467-019-09234-6

5. Zhang B, Horvath S. A general framework for weighted gene co-expression network analysis. *Stat Appl Genet Mol Biol*. 2005;4:Article17. doi: 10.2202/1544-6115.1128

6. Theofilatos K, Stojkovic S, Hasman M, van der Laan SW, Baig F, Barallobre-Barreiro J, Schmidt LE, Yin S, Yin X, Burnap S, et al. Proteomic Atlas of Atherosclerosis: The Contribution of Proteoglycans to Sex Differences, Plaque Phenotypes, and Outcomes. *Circ Res*. 2023;133:542-558. doi: 10.1161/circresaha.123.322590

7. Herrington DM, Mao C, Parker SJ, Fu Z, Yu G, Chen L, Venkatraman V, Fu Y, Wang Y, Howard TD, et al. Proteomic Architecture of Human Coronary and Aortic Atherosclerosis. *Circulation*. 2018;137:2741-2756. doi: 10.1161/CIRCULATIONAHA.118.034365

8. Nehme A, Kobeissy F, Zhao J, Zhu R, Feugier P, Mechref Y, Zibara K. Functional pathways associated with human carotid atheroma: a proteomics analysis. *Hypertens Res*. 2019;42:362-373. doi: 10.1038/s41440-018-0192-4

9. Vaisar T, Hu JH, Airhart N, Fox K, Heinecke J, Nicosia RF, Kohler T, Potter ZE, Simon GM, Dix MM, et al. Parallel Murine and Human Plaque Proteomics Reveals Pathways of Plaque Rupture. *Circ Res*. 2020;127:997-1022. doi: 10.1161/CIRCRESAHA.120.317295
